# Resistance of *Mycobacterium tuberculosis* to indole 4-carboxamides occurs through alterations in drug metabolism and tryptophan biosynthesis

**DOI:** 10.1016/j.chembiol.2021.02.023

**Published:** 2021-08-19

**Authors:** M. Daben J. Libardo, Caroline J. Duncombe, Simon R. Green, Paul G. Wyatt, Stephen Thompson, Peter C. Ray, Thomas R. Ioerger, Sangmi Oh, Michael B. Goodwin, Helena I.M. Boshoff, Clifton E. Barry

**Affiliations:** 1 Tuberculosis Research Section, Laboratory of Clinical Immunology & Microbiology, National Institute of Allergy and Infectious Diseases, National Institutes of Health, Bethesda, MD 20892, USA; 2 Drug Discovery Unit, Division of Biological Chemistry and Drug Discovery, School of Life Sciences, University of Dundee, Dundee DD1 5EH, UK; 3 Department of Computer Science and Engineering, Texas A&M University, College Station, TX 77843, USA; 4 Institute for Infectious Disease and Molecular Medicine, University of Cape Town, Cape Town 7935, South Africa

**Keywords:** tuberculosis, drug mechanism of action, pro-drug, tryptophan metabolism, antimetabolite

## Abstract

Tryptophan biosynthesis represents an important potential drug target for new anti-TB drugs. We identified a series of indole-4-carboxamides with potent antitubercular activity. *In vitro*, *Mycobacterium tuberculosis* (Mtb) acquired resistance to these compounds through three discrete mechanisms: (1) a decrease in drug metabolism via loss-of-function mutations in the amidase that hydrolyses these carboxamides, (2) an increased biosynthetic rate of tryptophan precursors via loss of allosteric feedback inhibition of anthranilate synthase (TrpE), and (3) mutation of tryptophan synthase (TrpAB) that decreased incorporation of 4-aminoindole into 4-aminotryptophan. Thus, these indole-4-carboxamides act as prodrugs of a tryptophan antimetabolite, 4-aminoindole.

## Highlights

• *Mtb* is inhibited by indole-4-carboxamides-exogenous tryptophan intermediates rescue
• Mutations in *amiC* result in decreased hydrolysis of indole-4-carboxamides
• *trpE* mutants lose feedback inhibition by L-Trp increasing flux through the pathway
• 4-Aminoindole is incorporated into 4-amino-L-tryptophan poisoning cellular metabolism

## Graphical abstract

**Figure:**
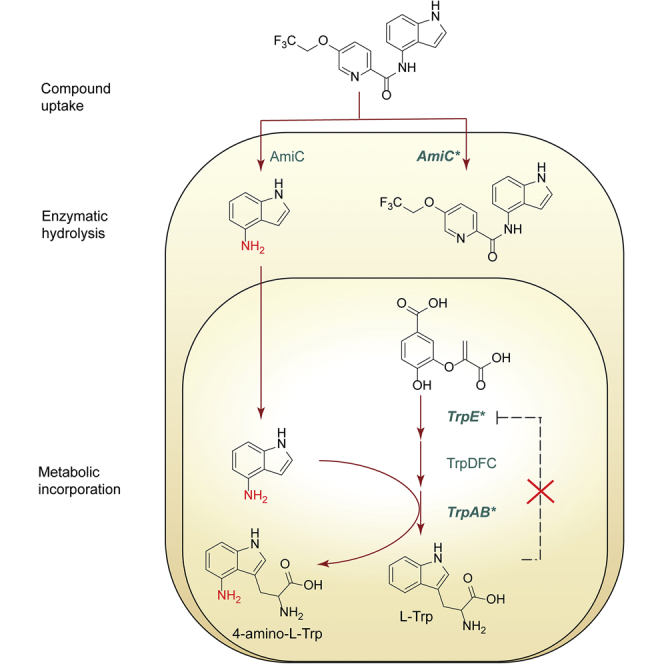

## Teaser

Libardo et al. demonstrate that indole-4-carboxamides are cleaved by an intracellular amidase of *Mycobacterium tuberculosis* liberating 4-aminoindole that is subsequently converted into toxic 4-amino-L-tryptophan by tryptophan synthase. Mutation in the amidase and two enzymes involved in tryptophan biogenesis confer resistance and illuminate the complexity of metabolic control of tryptophan levels.

## Introduction

*Mycobacterium tuberculosis* (Mtb), the causative agent of tuberculosis (TB), continues to take its toll on humanity with about 7 million new infections and 1.5 million people succumbing to the disease in 2018 (WHO, 2020). While the outlook looks dire with the rise of strains resistant to one or more of the clinically approved drugs for TB, several new compounds with novel targets have reached clinical trials (Libardo et al., 2018), and treatment shortening regimens are being developed (Lee et al., 2020), offering hope to patients. The rise of multidrug-resistant Mtb reflects a vast repertoire of mechanisms the bacillus deploys to subvert drug action—from target modification to drug efflux and degradation (Mabhula and Singh, 2019). A thorough understanding of a drug's mechanism of action and the ways in which Mtb resist their cidal effects is paramount to developing next-generation antitubercular compounds.

**Figure 1:**
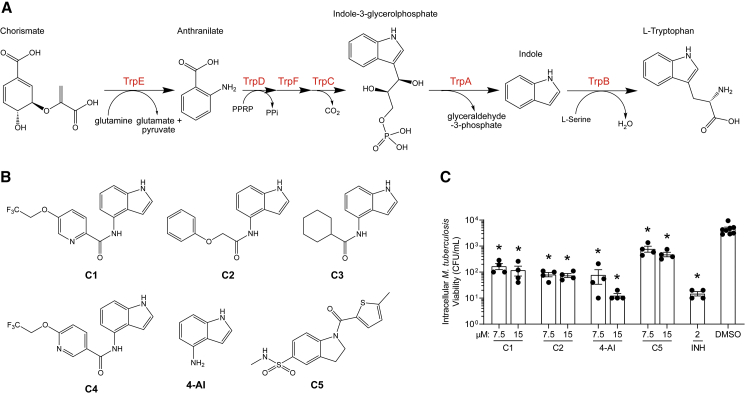
Antimycobacterial indole-4-carboxamides (A) The tryptophan biosynthesis pathway and the enzymes (in red) catalyzing each transformation. (B) Structures of the compounds used in this study. (C) J774 macrophages were infected with Mtb H37Rv (multiplicity of infection = 1:10) and treated with the corresponding compound at the indicated concentrations for 7 days. The macrophages were lysed and surviving Mtb were enumerated by plating multiple dilutions in 7H11 plates. Limit of detection = 10 CFU/mL. Bars represent mean ± SEM (n = 4). ∗p < 0.001 using one-way ANOVA.

Tryptophan biosynthesis represents a promising pathway to target with novel drugs. Chorismate from the shikimate pathway is converted to L-tryptophan (L-Trp) in six enzymatic steps (Figure 1A). The first committed step in the biosynthesis is catalyzed by anthranilate synthase (TrpE). Anthranilate is converted to indole-3-glycerolphosphate by the actions of TrpD, TrpF, and TrpC. The heterotetrameric tryptophan synthase (TrpAB) complex catalyzes the last two steps; the α subunit splits indole-3-glycerolphosphate (IGP) to indole and glyceraldehyde-3-phosphate (G3P) and the β subunit condenses indole with L-serine (L-Ser) to form L-Trp. Due to the high energetic cost associated with the biosynthesis of L-Trp, this pathway is regulated both transcriptionally (Merino et al., 2008) and allosterically by L-Trp (Calhoun et al., 1973). Mtb that were made auxotrophic for L-Trp by deletion of either *trpE* or *trpA* failed to persist in axenic culture and were avirulent in mice (Wellington et al., 2017; Zhang et al., 2013), establishing the essentiality of the pathway to the pathogenesis of Mtb. On the host side, interferon-γ-activated macrophages induce expression of indoleamine-2,3-dioxygenase-1 (IDO-1), which leads to the conversion of L-Trp to kynerunine, a process that inhibits the growth of intracellular pathogens with L-Trp auxotrophy (Ibana et al., 2011). This strategy, however, is not effective against Mtb and other bacteria capable of *de novo* L-Trp biosynthesis. Nevertheless, catabolism of L-Trp by IDO-1 was evident in plasma samples obtained from patients with either active or latent TB (Adu-Gyamfi et al., 2020; Collins et al., 2020), further implicating the importance of L-Trp biosynthesis in Mtb infection. Indeed, chemical inhibition of bacterial L-Trp biosynthesis was shown to synergize with the host-induced L-Trp starvation to afford Mtb killing *in vivo* (Zhang et al., 2013), demonstrating the feasibility of targeting this pathway for Mtb chemotherapy.

Several recent studies established the susceptibility to pharmacological control of this essential mycobacterial pathway by multiple scaffolds. First, an azetidine (BRD4592), a sulfolane, and an indoline-5-sulfonamide all tightly bind to the interfacial region of TrpAB, stabilizing the closed, active state of the complex and inhibiting both subunits of the enzyme (Abrahams et al., 2017; Michalska et al., 2020; Wellington et al., 2017). Second, indole propionic acid mimics L-Trp and inhibits TrpE by binding to the allosteric L-Trp binding site (Negatu et al., 2019). Third, benzoate-based compounds bind to the TrpD active site and competitively inhibit the phosphoribosyl transferase activity (Castell et al., 2013; Evans et al., 2014). Finally, fluoroanthranilates, rather than inhibiting a discrete enzyme in the pathway, are utilized as a substrate to form fluorotryptophans, which are proposed to inhibit the growth of Mtb (Nurul Islam et al., 2019).

Here, we report a series of antimycobacterial indole-4-carboxamide prodrugs that liberate 4-aminoindole, which inhibits neither TrpE nor TrpAB, but rather is metabolically incorporated to form 4-aminotryptophan. We uncovered resistance mechanisms that illustrate how modulation of metabolic flux effectively rescues Mtb from the toxic incorporation of 4-aminoindole.

## Results

### Mutants resistant to indole-4-carboxamides clustered into two distinct groups

A series of small molecules consisting of carboxamidated indoleamine derivatives were obtained from an initial hit compound identified from a high-throughput whole-cell screen for inhibitors of Mtb growth. We studied the antimycobacterial properties and the mechanism of action of representative members of the series; compounds C1 through C4 (Figure 1B). All four compounds were found to have potent activity, inhibiting growth of both Mtb and *M. bovis* BCG at low μM concentrations in BSA-free media (Tables 1 and S1). Furthermore, C1 was not toxic to J774 macrophages (Figure S1) and afforded a 1.5 log reduction in intracellular Mtb titer when tested in a murine macrophage model of infection (Figure 1C).

**Table 1:** Minimum inhibitory concentration of compounds presented in this study in BSA-free medium (7H9/glucose/casitone/tyloxapol)

Strain	MIC (μM)							Mutation
	C1	C2	C3	C4	4-AI	C5	INH	
WT	1.56	3.12	6.25	3.12	4.68	0.39	0.12	–
5A	>100	75	>100	75	4.68	0.39	0.12	*amiC* K195∗
5B	>100	75	>100	75	4.68	0.39	0.12	*amiC* W238∗
5G	>100	75	>100	75	4.68	0.39	0.12	*amiC* S157F
5H	>100	75	>100	75	4.68	0.39	0.12	*amiC* + g in aa352
5I	>100	75	>100	75	4.68	0.39	0.12	*amiC* + g in aa352
5J	6.25	25	>100	25	100	0.39	0.12	*trpB* A168V
5K	6.25	25	>100	25	100	0.39	0.12	*trpB* A134V
5L	6.25	25	>100	25	100	0.39	0.12	*trpE* H170R
5M	6.25	25	>100	25	100	0.098	0.12	*trpB* D261G
5N	6.25	25	>100	25	100	0.19	0.12	*trpA* D54G
5O	6.25	25	>100	25	100	0.19	0.12	*trpB* D261G

To determine the mechanism of action, we isolated resistant mutants of *M. tuberculosis* on solid medium containing C1 at five times the *in vitro* MIC (minimum inhibitory concentration). Spontaneous resistance was observed at frequencies that varied depending on the number of bacilli plated; that is, when 10^7^ CFU (colony-forming unit) was plated, the frequency of resistance (FoR) was ~3 × 10^−6^, whereas at higher cell densities (10^9^ CFU), the FoR was ~3 × 10^−8^. Determination of C1 MICs against these mutants revealed high-level (>12-fold) resistance from the first group of mutants (5A-5I) and intermediate-level (4- to 8-fold) resistance in the latter group (5J-5O) (Table 1). These mutants were not only cross-resistant to C2, C3, and C4, but also exhibited a level of resistance similar to that seen with C1, indicating that the four compounds likely have identical mechanisms. The congruence between FoR and MICs suggested distinct mechanisms of resistance between the two mutant groups.

Whole-genome sequencing revealed two genomic loci whose single-nucleotide polymorphisms (SNPs) potentially conferred resistance against C1 (Table 1). The mutants that appeared at high FoR with lower bacterial numbers were found to be associated with mutations in a non-essential, putative amidase AmiC (*rv2888c*). On the other hand, the low FoR mutants that appeared with higher cell density had missense mutations in either anthranilate synthase subunit I (TrpE, *rv1609*), or the α or β subunit of tryptophan synthase (TrpA, *rv1613*; or TrpB, *rv1612*), all of which are essential enzymes in the tryptophan biosynthetic pathway (Wellington et al., 2017; Zhang et al., 2013).

### AmiC mutations abolishes degradation of C1

**Figure 2:**
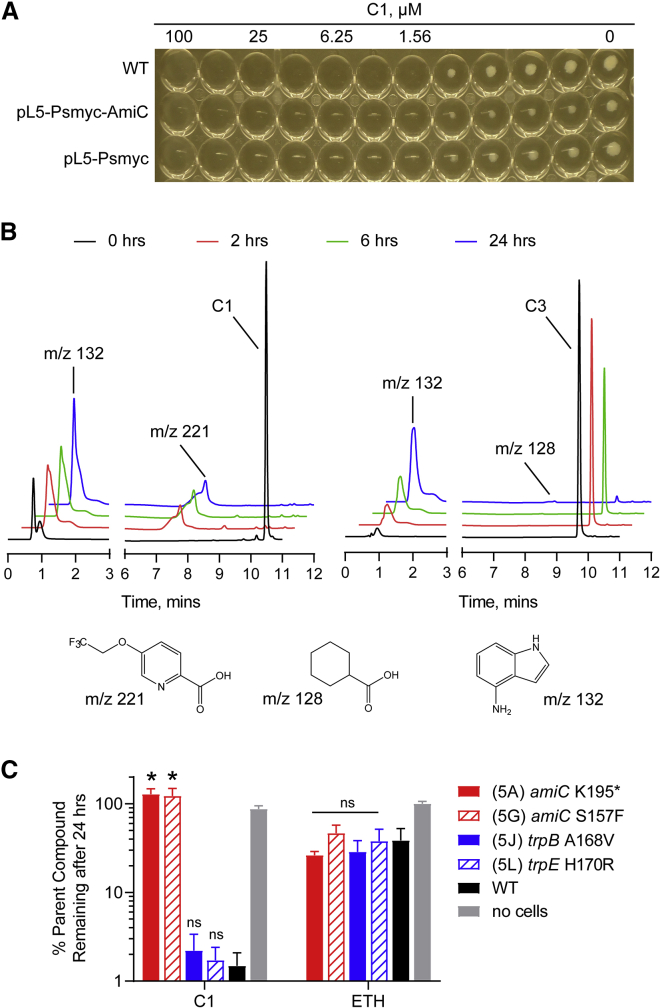
Loss-of-function mutations in AmiC result in decreased hydrolysis of indole-4-carboxamides (A) AmiC was overexpressed in H37Rv by introducing an extrachromosomal copy of *rv2888c* under the control of the P_smyc_ promoter (see STAR methods for details). The MIC of the WT and the overexpressing strains were measured using a standard broth microdilution method in BSA-free media. Results shown are representative of three independent trials. (B) To study Mtb-promoted compound metabolism, log phase Mtb cells (OD_600_ = 3.0) were exposed to 10× MIC of C1 (left) or C3 (right) in PBS and small aliquots were withdrawn, and quenched with acetonitrile and then injected into the LC-MS. The chromatograms shown are representative of three independent trials. Structures of the proposed degradation product are shown below with the corresponding m/z values. The retention time and mass were confirmed compared with the commercially available acid. (C) Mtb-dependent degradation of C1 was monitored in representative resistant mutants using a similar LC-MS-based assay as described above. Values were calculated as percent C1 remaining relative to the concentration present at 0 h. ETH, ethionamide. Bars represent mean ± SEM (n = 3). ∗p < 0.001; ns, not significant when compared with the corresponding WT run using one-way ANOVA.

The homolog of *amiC* in *M. smegmatis* (Msm) (*msmeg_2521*) was shown to function as an inducer of acetamidase expression to allow growth using amides as a sole carbon source (Roberts et al., 2003). While Msm AmiC is only 65% identical to AmiC in Mtb, one of the residues that was mutated (Ser157) in the *amiC* mutants is highly conserved in several mycobacterial species. The non-essential nature of *amiC* in Mtb coupled with its putative function led us to speculate that it was involved in C1 metabolism. Overexpression of AmiC, via introduction of an extrachromosomal copy of *rv2888c* under the control of the strong mycobacterial promoter (P_smyc_) (Ehrt et al., 2005), did not result in a noticeable change in MIC against C1 (Figure 2A), suggesting that AmiC was not the direct target of C1.

We next incubated the compounds with live Mtb and monitored the levels of the parent amides via liquid chromatography-mass spectrometry (LC-MS). We observed complete disappearance of the parent compound peak from C1 after 2 h with a concomitant appearance of a peak with an m/z corresponding to the free carboxylic acid (Figure 2B). The m/z of the free indoleamine was also found, eluting in the void volume. The hydrolysis of C1 proceeding to completion with wild-type (WT) levels of AmiC also explains why overexpression of this protein did not decrease the MIC. In addition, the rate of degradation seemed to be correlated with MICs, as C1 was completely metabolized within 2 h, whereas the less-active derivative C3 showed residual levels of the amide after 24 h (Figure 2B). To establish drug metabolism in the various resistant strains we had isolated, we performed the same assay in representative mutants. We found that the *amiC* mutants had lost the ability to metabolize C1 while mutants in L-Trp biosynthesis had no effect on metabolism (Figure 2C). This suggested that the *amiC* mutations were loss-of-function mutations that prevented hydrolysis of the amides. Whether AmiC directly hydrolyses C1 or regulates the expression of other amidases—like its homolog in Msm—was not explored in this study. We note, however, that compounds C1 through C4 were all inactive in *M. smegmatis* (Table S1).

The fact that *amiC* mutations resulting in abrogated amide hydrolysis leads to high-level resistance also suggests that C1 is a pro-drug liberating an active metabolite from amide hydrolysis. We therefore determined whether the amine or the carboxylic acid that would result from hydrolysis of C1 were responsible for the observed activity. All four representative compounds share the 4-aminoindole (4-AI) moiety but differ in the carboxylic acid coupling partner. Measuring the MIC of these building blocks showed that only 4-AI was active against Mtb (MIC = 4.68 μM), while the free acids had little to no potency (Table S2). In addition, members of this compound series in which the carboxamide linkage is altered (either via N-methylation, replacement with a secondary amine linker, or flipped such that the indole carries the carboxylic acid) were all inactive against Mtb (compounds C10–C13 in Table S3), further supporting that 4-AI was responsible for the observed activity. Unsurprisingly, the *amiC* mutants were not cross-resistant to 4-AI (a molecule that bypasses the AmiC hydrolysis requirement), while the *trpABE* mutants were highly resistant to it (Table 1), indicating that 4-AI derived from the AmiC-mediated hydrolysis of indole-4-carboxamides likely targets L-Trp biosynthesis.

### 4-AI induce perturbations in tryptophan biosynthesis

**Figure 3:**
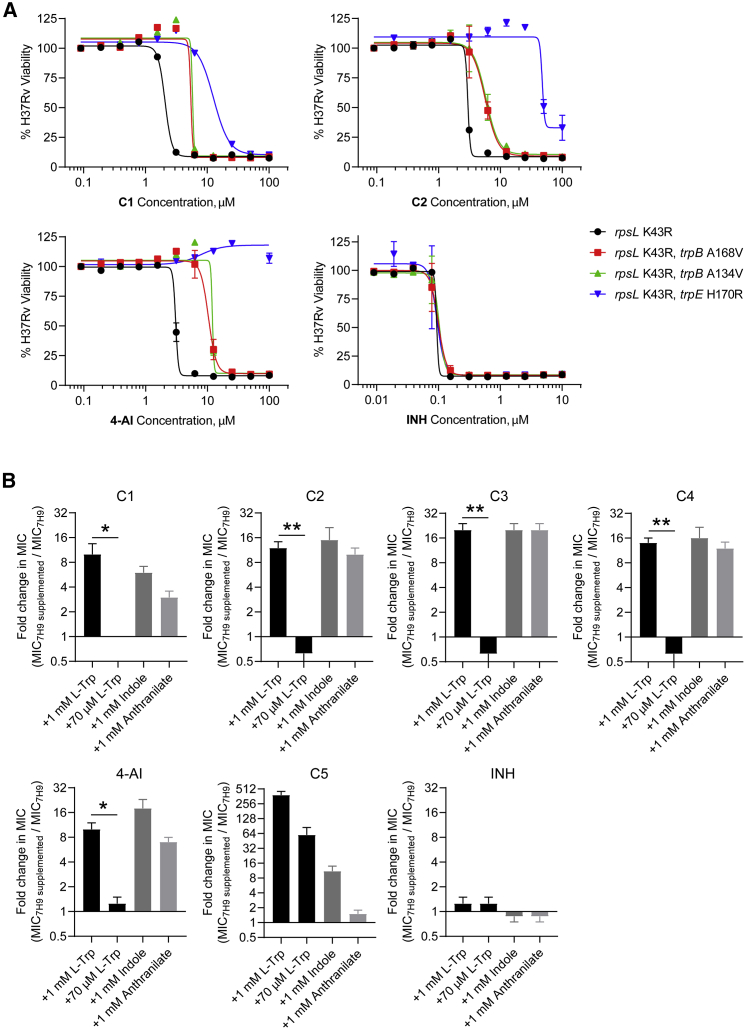
Indole-4-carboxamides induce perturbations in tryptophan biosynthesis (A) Native copies of *trpB* and *trpE* in H37Rv were replaced with mutant alleles identified from resistant mutants via single-stranded DNA-mediated recombineering. The activity of the compounds was tested using a resazurin microtiter assay and presented as percent viability relative to untreated cells. INH, isoniazid. (B) The MICs of the compounds were measured with or without supplementation of the medium with the indicated metabolite. Bars represent mean ± SEM (n = 4). ∗p < 0.05, ∗∗p < 0.005 using unpaired t test.

Having established the role of AmiC in the activity of C1, we set out to study the effect of C1 and 4-AI on tryptophan biosynthesis. We first confirmed that the SNPs in the tryptophan biosynthetic genes were resistance conferring by reintroducing a subset of the mutations to a WT Mtb H37Rv background. We electroporated Mtb with single-stranded oligonucleotides to replace the native copies of *trpB* or *trpE* with the mutant alleles by cellular recombineering (Murphy et al., 2015). We co-electroporated the cells with an oligo that would introduce the RpsL K43R mutation to allow for selection of recombinants using streptomycin (Spies et al., 2008). We found that the recombineered strains were resistant to C1, C2, and 4-AI but not to isoniazid (INH) (Figure 3A), recapitulating our mutant MIC results and establishing that these mutations were indeed responsible for resistance.

Supplementing the growth medium with 1 mM of the products of TrpA (indole), TrpB (L-Trp), or TrpE (anthranilate) rescued Mtb from the inhibitory activity of the indole-4-carboxamides and 4-AI, increasing MICs 4- to 16-fold (Figure 3B). Interestingly, addition of lower amounts of L-Trp (70 μM) had no effect on the MIC. The ability of various primary metabolites to rescue Mtb from growth inhibition by these compounds suggests that 4-AI is likely exerting a dominant pleiotropic effect on tryptophan biosynthesis that is only overridden at high concentrations of L-Trp. As a control, we utilized C5—an indoline-5-sulfonamide compound (Figure 1B) that was previously reported to selectively inhibit mycobacterial TrpAB (Abrahams et al., 2017). C5 potency was significantly attenuated by exogenous indole or L-Trp (even at 70 μM) but not by anthranilate; the result one would expect from a compound like C5 that inhibits the final step of the pathway. We therefore inferred that the indole-4-carboxamides and 4-AI were unlikely to be acting as direct inhibitors of TrpAB.

### Mutations in TrpE block feedback inhibition and increase precursors of the pathway

**Figure 4:**
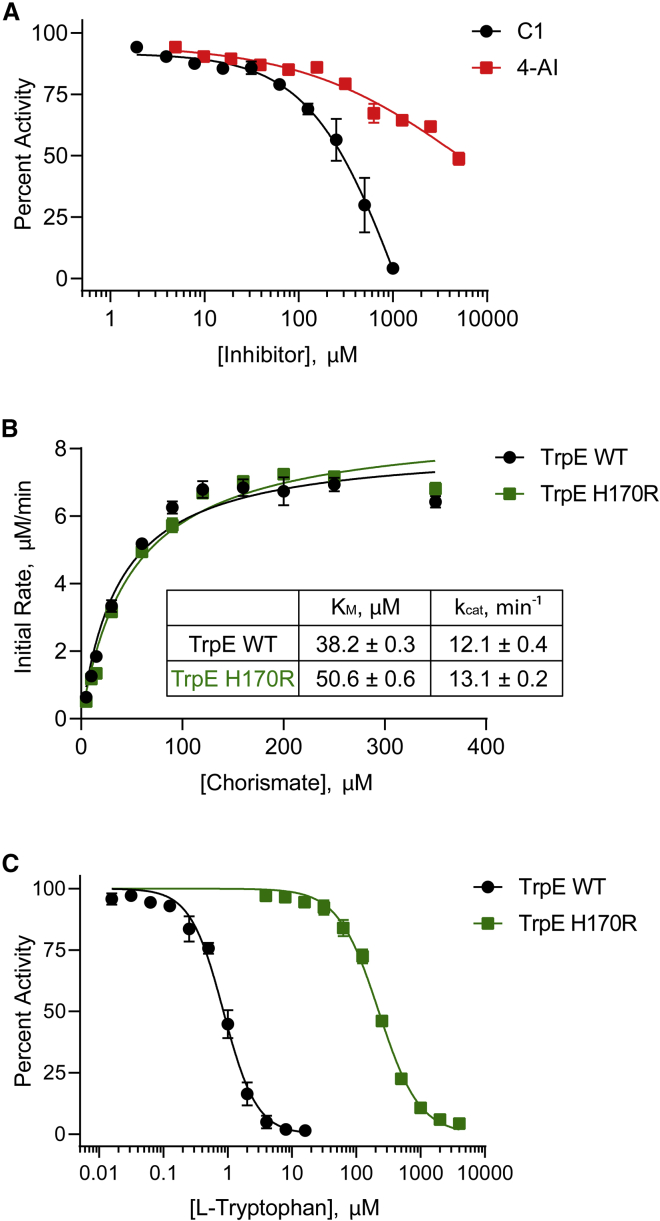
Resistance due to the TrpE H170R mutation results from loss of allosteric feedback inhibition (A) Inhibition of TrpE by C1 and 4-AI was studied in the presence of saturating concentrations of chorismate (50 μM) and NH_4_^+^ (100 mM). Inhibition is reported as percent activity relative to the no inhibitor control. (B) Steady-state kinetics of WT TrpE and the H170R variant were studied under varying chorismate concentrations by directly monitoring formation of the fluorescent product, anthanilate. (C) Allosteric feedback inhibition of TrpE WT and the H170R mutant was measured under increasing concentrations of L-Trp and presented as percent activity relative to the no L-Trp control. Data in all panels represent mean ± SEM (n = 3).

The TrpE H170R mutant protein resulted in an approximately 4-fold shift in MIC for C1-C4 and 4-AI. To study the interaction of these compounds with TrpE, we expressed and purified both the TrpE WT and the TrpE H170R mutant proteins. An *in vitro* enzyme assay showed that neither 4-AI (half-maximal inhibitory concentration [IC_50_] = 6.2 ± 1.4 mM) nor C1 (IC_50_ = 242 ± 53 μM) inhibited TrpE at concentrations near their MICs (Figure 4A), indicating that TrpE is not directly targeted by these compounds. We compared the kinetic parameters of the WT with the H170R mutant and found that, while a small change in chorismate K_M_ was found, it did not lead to a noticeable change in catalytic efficiency, k_cat_ (Figure 4B).

The crystal structure of Mtb TrpE shows that His170 is buried in the TrpE homodimer interface and interacts with several residues in the allosteric loop within the L-Trp binding site (Bashiri et al., 2015). Previous studies have also shown that mutations in the allosteric L-Trp binding site of TrpE lead to resistance against fluoroanthranilates and indole propionic acid (Negatu et al., 2019; Zhang et al., 2013), which, like 4-AI, are structurally similar to the intermediates of L-Trp biosynthesis. When we assayed the inhibition of TrpE, we found that the H170R mutant (IC_50_ = 221 ± 12 μM) was 250 times less sensitive to allosteric inhibition by L-Trp than WT enzyme (IC_50_ = 0.87 ± 0.11 μM) (Figure 4C). This suggests that the H170R mutation makes TrpE refractory to feedback inhibition by L-Trp, which normally functions to restrict flow of metabolites through the pathway. Therefore, this mutation in TrpE enhances the flux of intermediates through the pathway at L-Trp concentrations that would otherwise shut down biosynthesis, allowing Mtb to overcome inhibition by 4-AI.

### Discrete mutations in TrpAB determine the resistance profile

**Figure 5:**
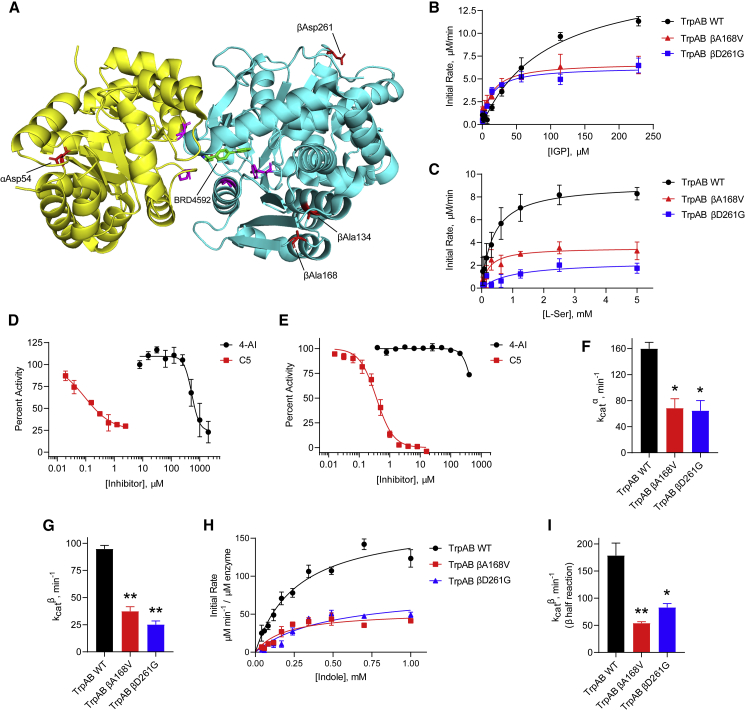
Resistance due to TrpAB mutations result from attenuation of enzymatic activity (A) 3D crystal structure of Mtb TrpAB with bound inhibitor (BRD4592, PDB: 5TCI) showing the location of the resistance-conferring mutations. The α subunit is shown in yellow, the β subunit in cyan. Mutations in C1^R^ strains are shown in red, while mutations in C5^R^ strains are shown as magenta. (B and C) Fluorometric determination of the steady-state kinetics of WT and mutant TrpAB (IGP + L-Ser → G3P + L-Trp) under saturating L-Ser (B) or IGP (C) concentrations. Data shown are mean ± SEM (n = 3). (D) Inhibition of the TrpAB α reaction was monitored by coupling the IGP consumption to the spectrophotometric detection of the liberated G3P. Data are mean ± SEM (n = 2). (E) Inhibition of the TrpAB β reaction (using indole as substrate) measured by monitoring the change in absorbance due to formation of L-Trp. Data represent mean ± SEM (n = 3). (F and G) Catalytic efficiency, k_cat_, of TrpAB α subunit (F) and β subunit (G) in the WT and mutant enzymes calculated from the Michaelis-Menten curves in (B) and (C), respectively. Bars represent mean ± SEM (n = 3). ∗p < 0.05, ∗∗p < 0.001 when compared with the corresponding WT k_cat_ using one-way ANOVA. (H) Spectrophotometric determination of the kinetics of the β half-reaction (indole + L-Ser → L-Trp) of the WT (50 nM) and mutant (200 nM) TrpAB. Data are mean ± SEM (n = 2). (I) Calculated catalytic efficiencies for the β half-reaction of the WT and mutant TrpAB from (H). Bars represent mean ± SEM (n = 2). ∗p < 0.05, ∗∗p < 0.01 when compared with the WT k_cat_ using one-way ANOVA.

None of the resistant mutants raised against C1 were cross-resistant to C5 (Table 1); in fact, one C1^R^ mutant was more susceptible to C5 relative to WT, suggesting a potential synergistic effect. We independently raised resistant mutants against C5 and, in agreement with previously published results (Abrahams et al., 2017), we also found mutations in TrpAB (αP65L, αD136N, βF188S, and βY200C). In a reciprocal experiment measuring the MIC of C1 and 4-AI against the C5^R^ mutants, no cross-resistance was observed (Table S4). Taken together, these results demonstrate that, while independent TrpAB mutations confer resistance to either C1 or C5, these two compounds have distinct mechanisms of action. Substantiating this claim is a map of where the mutated residues lie in the 3D structure of TrpAB. The mutations that conferred resistance to C5 were found to cluster in the interface between the α and β subunits, as reported for indolinesulfonamide-based (Abrahams et al., 2017) and azetidine-based (Wellington et al., 2017) inhibitors that bind tightly to this region. On the other hand, mutated residues conferring resistance to C1 and 4-AI were scattered throughout the protein structure (Figure 5A). Notably, βAla134 and βAla168 are located in helix 4 and strand 7, respectively, within the β subunit communications domain—a region of the complex that mediates intersubunit allosteric cooperativity (Dunn, 2012). In the α^o^β^c^ inhibitor-bound structure of Mtb TrpAB (PDB: 5TCI), αAsp54 is >22 Ǻ away from the α subunit catalytic residue αAsp68 (Bahar and Jernigan, 1999), while all TrpB lesions are >10 Ǻ away from the catalytic pyridoxal 5′-phosphate cofactor. The locations of the mutated residues easily explain the lack of cross-resistance of the C1^R^ mutants to C5, and vice versa. Furthermore, the dispersed nature of the TrpAB-mutated residues in the C1^R^ mutants, along with their sheer distance from the subunit active sites, and the fact that a specific TrpE lesion also confers resistance, all suggest that the activity of 4-AI may not be based on binding to and inhibition of a single enzyme. To confirm this, we expressed and purified enzymatically active forms of both WT and mutant TrpAB and studied their kinetics *in vitro*.

### TrpAB mutants that confer resistance to 4-AI have attenuated enzymatic activity

To study the kinetics of the TrpAB complex, we utilized a coupled enzyme assay that detects the formation of G3P and measured the kinetic parameters under saturating IGP or L-Ser concentrations. We calculated an apparent K_M_^IGP^ = 76.9 ± 1.1 μM (Figure 5B), and an apparent K_M_^L−Ser^ = 0.55 ± 0.23 mM (Figure 5C), in agreement with previously reported values (Wellington et al., 2017); and subunit catalytic efficiencies, k_cat_^α^ = 159.8 ± 9.7 min^−1^ and k_cat_^β^ = 94.8 ± 3.3 min^−1^, indicating that the recombinant enzyme was catalytically active. We found that 4-AI did not inhibit either subunit (IC_50_^α^ = 630.7 ± 173.8 μM and IC_50_^β^ = 649.4 ± 2.5 μM) at concentrations as low as that of the bona fide TrpAB inhibitor C5 (IC_50_^α^ = 0.10 ± 0.06 μM and IC_50_^β^ = 0.37 ± 0.07 μM) (Figures 5D and 5E), confirming that the 4-AI whole-cell activity was not due to inhibition of the final steps of L-Trp biosynthesis.

We studied the effect of representative mutations on the enzymatic activity of TrpAB to unravel the molecular basis of resistance (Figures 5B and 5C). We selected the βA168V mutant because this residue is located in the COMM domain—known to coordinate catalysis in both subunits—and the βD261G mutant, since this residue is located in an inconspicuous, solvent-exposed region of the complex that is likely serving a structural purpose. We find that both mutations effectively decrease the catalytic activity of both the α and β subunits to 35%–45% of the WT (Figures 5F and 5G) when IGP was used as a substrate. In addition, looking exclusively at the half-reaction catalyzed by the β subunit (indole + L-Ser → L-Trp), we observed the catalytic efficiency dropping 3-fold in the mutant enzymes (Figures 5H and 5I). This suggested that attenuation of enzymatic activity might result in evasion of the toxicity of 4-AI. This result is in contrast to canonical resistance mechanisms against specific enzyme inhibitors, which typically involve mutations resulting in either overexpression of the target (Larsen et al., 2002; Park et al., 2017) or modification of the binding site (Schaenzer and Wright, 2020), both of which compensate for the effect of the inhibitor by maintaining the concentration of the enzymatic product to homeostatic levels.

### 4-AI is converted to 4-amino-L-Trp by TrpB

**Figure 6:**
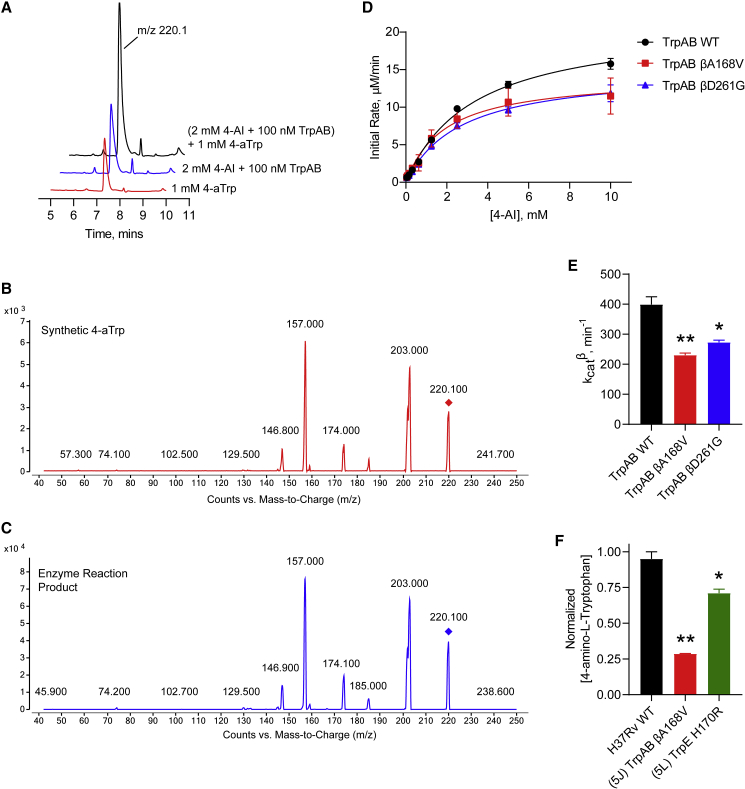
TrpB converts 4-AI to 4-amino-L-Trp *in vitro* and in Mtb (A) Representative chromatograms of synthetic 4-aTrp (red trace), the enzymatic reaction mixture obtained by incubating TrpAB with 4-AI (blue trace), and the enzyme reaction spiked with synthetic 4-aTrp (black trace). (B and C) Fragmentation pattern of synthetic 4-aTrp (B) and the enzyme reaction mixture (C) in a triple quadrupole mass spectrometer. (D) 4-AI was incubated with WT or mutant TrpAB in the presence of saturating L-Ser concentrations. The resulting 4-aTrp was quantified using LC-MS. Data shown are mean ± SEM (n = 2). (E) Calculated catalytic efficiency, k_cat_, of the WT and mutant TrpAB in (D). Bars represent mean ± SEM (n = 2). ∗p < 0.05, ∗∗p < 0.01 when compared with the WT k_cat_ using one-way ANOVA. (F) Intracellular 4-AI incorporation into 4-aTrp was measured by extracting the metabolites using 1:1 ACN:MeOH. The concentration of 4-aTrp produced within the indicated C1^R^ mutants were normalized against that of the WT. Bars represent mean ± SEM (n = 2). ∗p < 0.05, ∗∗p < 0.01 when compared with the WT using one-way ANOVA.

Motivated by the lack of enzymatic inhibition, and the fact that 4-AI is an analog of indole, we explored the possibility of 4-AI being incorporated into L-Trp biosynthesis by TrpB. We incubated purified TrpAB with 4-AI instead of indole under saturating L-Ser concentrations and observed the appearance of a product peak with an m/z of 220, which we hypothesized was 4-amino-L-Trp (4-aTrp) (Figure S2). To confirm that this new product was indeed 4-aTrp, we synthesized an authentic 4-aTrp standard by nitrating L-tryptophan and separating the minor 4-nitro isomer after protection of the tryptophan amino group with a t-butoxycarbonyl group (Figure S3). Following nitro reduction and deprotection we obtained authentic 4-aminotryptophan and ran an LC-MS co-elution assay (Figure 6A). We found that the chemically synthesized 4-aTrp eluted at a retention time identical to that of the product of the enzymatic reaction. In addition, spiking the enzyme reaction with the synthetic 4-aTrp demonstrated co-elution. Furthermore, the fragmentation pattern of the enzymatic product was indistinguishable from that of the chemical standard under various collision-induced dissociation potentials (Figures 6B, 6C, S4A, and S4B). Together, these results indicate that the product peak observed upon feeding TrpAB with 4-AI indeed corresponds to 4-aTrp, and that TrpB is capable of utilizing 4-AI as a substrate.

To study the kinetics of 4-AI utilization by the WT and the mutant TrpAB, we incubated the recombinant enzymes with increasing 4-AI concentrations (under a saturating L-Ser concentration) for 5 min, quenched the reaction with 0.1% formic acid in methanol, and detected the formation of 4-aTrp via LC-MS. The k_cat_ values for the β reaction for the WT enzyme with 4-AI (Figure 6E) appeared to be about twice that of the corresponding β reaction for the native indole substrate (Figure 5I). We found that both TrpAB mutants utilize 4-AI less efficiently as a substrate compared with the WT enzyme (Figures 6D and 6E), similar to the effect observed when indole is used as a substrate (Figure 5I). Therefore, these mutations decrease the incorporation of 4-AI, potentially reducing the toxicity of the resulting 4-aTrp and imparting resistance to the cell. In a scenario where TrpB uses 4-AI, the cell would require higher levels of exogenous L-Trp to minimize and compete with the buildup of endogenous 4-aTrp, retrospectively explaining why 1 mM (but not 70 μM) of L-Trp was required to rescue the growth of Mtb in the presence of 4-AI (Figure 3B).

Finally, we incubated Mtb with 4-AI for 24 h under conditions that would promote incorporation and minimize cell death (10× MIC with Mtb at an optical density at 600 nm [OD_600_] = 3.0). We then extracted the metabolites in Mtb using 1:1 ACN:MeOH and injected aliquots in the LC-MS. We detected formation of 4-aTrp in WT cells (Figure S5) indicating that, in a cellular context, 4-AI indeed gets incorporated into the L-Trp biosynthetic pathway. The 4-aTrp concentration within the TrpAB βA168V-resistant mutant were significantly reduced compared with WT (Figure 6F) in agreement with the marked decrease in enzymatic catalysis we observed *in vitro*. Finally, in the TrpE H170R-resistant mutant, we detected 4-aTrp levels higher than in the TrpAB mutant but lower than in the WT. We hypothesized that 4-aTrp could also allosterically inhibit TrpE, restricting flow of metabolites and decreasing 4-AI incorporation, resulting in lower intracellular 4-aTrp concentration. However, when we measured the ability of 4-aTrp to affect allosteric inhibition of TrpE we found inhibition only with an IC_50_ value of 59.6 ± 4.5 μM that was not affected by mutations that alleviate allosteric regulation (IC_50_ versus TrpE H170R = 63.6 ± 8.3 μM) (Figure S6). While directly correlating the *in vitro* IC_50_ with the apparent IC_50_ against TrpE within Mtb cells is impossible, the relatively high *in vitro* IC_50_ suggests that 4-aTrp generated by TrpB is unlikely to feedback inhibit TrpE and shut down overall L-Trp biosynthesis. Exogenously added synthetic 4-aTrp did not have any antitubercular activity (MIC > 100 μM), but transport of this molecule into Mtb cells may not be very efficient. Incubating Mtb cells with 10 or 100 μM 4-AI over a 3-day period followed by detection of either L-Trp or 4-aTrp via LC-MS (Figure S7) showed that the intracellular L-Trp pool is unaffected by exogenous 4-AI. Furthermore, intracellular 4-aTrp levels rose to nearly ten times that of endogenous L-Trp levels, which remained under 5 μM over the same time period, indicating that the 4-AI cytotoxicity is likely due to intracellular accumulation of 4-aTrp that eventually gets incorporated into native proteins rather than L-Trp starvation. It should be noted that incorporation of fluorotryptophans into native proteins has been previously observed in *M. smegmatis* (Nurul Islam et al., 2019).

## Discussion

Our study showed three distinct mechanisms of Mtb resistance to indole 4-carboxamides. First, loss-of-function mutations in AmiC result in diminished hydrolysis of C1 and therefore no 4-AI—the active component—is liberated. In TB drug discovery, the shift to whole-cell screening followed by target deconvolution by whole-genome sequencing of resistors has identified many enzymes that metabolize drugs (Grant et al., 2016). Many of these “activating” enzymes are encoded by non-essential, dispensable genes resulting in high frequencies of resistance. Indeed, we picked up *amiC* mutations at a frequency of 10^−6^, 100-fold more common than the frequency of *trpABE* mutations. The frequency of resistance was dependent on the size of the inoculum; thus, when 10^9^ cells were plated, there were probably enough bacilli on the plate to hydrolyze all C1 to 4-AI, and we were inadvertently raising mutants against 4-AI. Whereas, when 10^7^ cells were plated, hydrolysis may not have been as extensive; hence, the mutants were selected against the parent compound C1. AmiC-mediated hydrolysis must have a certain degree of substrate promiscuity since derivatives that varied greatly in the size, polarity, and position of the carboxylic acid coupling partner relative to indoleamine displayed low μM MICs against Mtb (compounds C16-C21 in Table S3). It may also be possible that AmiC can mediate the hydrolysis of other potential antimycobacterial amides.

The second resistance mechanism we described here is a specific mutation in TrpE that makes the enzyme resistant to allosteric feedback inhibition by L-Trp—a phenomenon repeatedly observed in Mtb (Negatu et al., 2019; Zhang et al., 2013). This effectively keeps a steady flux of intermediates through the pathway under L-Trp concentrations that would normally shut down biosynthesis. A TrpE mutation that would prevent prematurely shutting down the entire pathway therefore will allow Mtb to produce enough L-Trp to a sufficient level to effectively compete with endogenous 4-aTrp for protein incorporation, overcoming the 4-AI-imposed L-Trp starvation. The results we obtained when the growth medium was supplemented with metabolic intermediates downstream of TrpE (Figure 3B) support the notion that maintaining or increasing the flux through the pathway will “dilute” the toxic effects of 4-aTrp buildup. Previously, 5-fluorotryptophan (produced from the incorporation of 5-fluoroanthranilate) was shown to also allosterically inhibit TrpE with an IC_50_ of 4 μM (Negatu et al., 2019)—an unsurprising result given the conservative nature of the hydrogen-to-fluorine substitution. Here, we show that the IC_50_ of 4-aTrp against TrpE is 15-fold higher, indicating that small alterations in the L-Trp structure can have a large effect in TrpE inhibition.

The final mechanism of resistance we uncovered in this study involves the alteration of TrpAB enzymatic activity that effectively decreases conversion of 4-AI to 4-aTrp while simultaneously decreasing the indole to L-Trp conversion as well. The TrpAB mutants grew equally well as the WT in rich media, indicating that the resistors had an exquisitely fine-tuned TrpAB activity, effectively lowering 4-AI incorporation while still satisfying nutritional requirements to maintain WT-like growth. The demonstration that, under homeostatic conditions, *de novo* L-Trp biosynthesis in Mtb only generates a small pool of L-Trp (cytosolic concentration <5 μM) (Agapova et al., 2019), coupled with the fact that Trp is one of the least-abundant amino acids in proteins (only accounting to ~1%) explains why even a weakened TrpAB is still able to meet the L-Trp demands of the cell. The TrpAB mutations we found were scattered throughout the protein structure, with two lesions located in the β subunit COMM domain. Throughout the catalytic cycle of TrpAB, the two subunits interact largely through the COMM domain (Dunn, 2012), which also creates part of the tunnel where indole produced from the α subunit is presumably shuttled through to reach the β subunit active site (Hilario et al., 2016). Therefore, it is expected that the βA168V mutation will result in faulty catalysis. On the other hand, why the βD261G mutation—a residue that lies in an inconspicuous location of the complex that has no annotated catalytic or cooperative function—leads to catalytic attenuation is still a mystery. We note that the βD261G mutation potentiates the activity of the validated TrpAB inhibitor (C5), rendering the mutant more susceptible to C5 compared with the WT (Table 1).

In this work, we describe a series of indole-4-carboxamides that, upon AmiC-mediated hydrolysis, liberate 4-AI, which gets incorporated into the L-Trp biosynthetic pathway to form 4-aTrp. The tolerance of the L-Trp biosynthetic enzymes in Mtb is demonstrated by a recent study that shows that fluoroanthranilates produce fluorotryptophans, but none of the fluorinated intermediates (Nurul Islam et al., 2019), indicating that all enzymes within the pathway accommodate minor substrate modifications and that fluorinated derivatives are incorporated through this pathway. Furthermore, the substrate promiscuity of TrpB (either isolated from other bacteria or optimized through directed evolution) has been exploited by chemists to enantioselectively synthesize a variety of L-Trp analogs that would otherwise be difficult to access (Buller et al., 2015; Romney et al., 2017; Winn et al., 2018). Small-molecule probes that harness the natural TrpB reactivity without significant cellular toxicity could, in theory, be used to monitor L-Trp biosynthesis in real time under various conditions and begin to answer why L-Trp starvation has a cidal effect in Mtb. It is logical to assume that various substituted L-Trp derivatives can also be incorporated into proteins via the action of equally promiscuous aminoacyl tRNA synthetases (Brustad et al., 2008); but how the structure and activity of these proteins will change in the presence of L-Trp analogs remains to be demonstrated. Biochemical analyses show that 4-aTrp in fluorescent proteins introduces significant pH sensitivity at the expense of protein stability (Budisa and Pal, 2004), and similar changes can be expected with mycobacterial proteins.

Even though TrpB can utilize an assortment of indole substitutions, the downstream effects of the substituted tryptophans in Mtb may vary greatly. For instance, positional isomers of C4 that would liberate 5- and 6-aminoindole were inactive, while those that liberate 7-aminoindole had only weak activity (compounds C6-C8 in Table S3). Similarly, 4-fluoroanthranilate was also more toxic to Mtb compared with the 5- and 6-substituted version (Nurul Islam et al., 2019). Furthermore, derivatives that would liberate 4-substituted N-methylindoles, indolines, or indazoles were all inactive against Mtb (compounds C9-C15 in Table S3). Therefore, there appears to be an inherent toxicity associated with L-Trp substituted at the 4-position. Complicating the situation is the possibility that toxicity may not be a direct result of incorporation into proteins. A global replacement of all L-Trp residues in a fluorescent protein found that, while 4- and 5-aTrp were quantitatively incorporated, there was no assimilation observed for 6- and 7-aTrp (Budisa et al., 2002).

In mycobacteria, previously demonstrated metabolic changes that confer drug resistance involve one of several mechanisms, including (1) overexpression of the target to overcome metabolic blockage by stoichiometrically limiting the number of enzymes deactivated by the drug (Park et al., 2017). (2) Using a compensatory pathway that bypasses the inhibited step while preserving the generation of the end product (Lamprecht et al., 2016). (3) Loss of function in an enzyme that catalyzes the opposing reaction of the target (Ballinger et al., 2019). A definitive demonstration of resistance acquisition through alteration of substrate recognition or enzymatic attenuation is lacking in the literature. In a similar sense, 5-fluorouracil (5-FU) was shown to be a metabolic poison in Mtb—being incorporated into cellular DNA and RNA (Singh et al., 2015). Single-nucleotide insertions in *rv3309*—encoding for one of the two uridine phosphoribosyltransferase in Mtb—was found in one group of 5-FU^R^ mutants. 5-FU was subsequently shown to be utilized by Rv3309 as a substrate in the biosynthesis of fluoro-UMP. Therefore, loss-of-function mutations in *rv3309* abrogated incorporation of 5-FU and resulted in resistance—a similar phenotype to that observed in our TrpAB mutants.

In conclusion, we demonstrated the mechanism of action of indole-4-carboxamides and the molecular basis of Mtb resistance against these compounds. We validated the idea that modulation of metabolism—either by maintaining flux through resistance from feedback inhibition (mutation in TrpE) or reducing flux through enzymatic attenuation (mutations in TrpAB)—affords antimicrobial resistance in Mtb. Poisoning tryptophan metabolism by deliberately engineering novel prodrugs of 4-AI with suitable pharmaceutical properties represents a promising avenue for the development of a novel antituberculosis agent.

## Significance

**With the rise of multidrug-resistant *Mycobacterium tuberculosis* (Mtb) comes the urgent need to identify potential new drug targets. Tryptophan biosynthesis has previously been identified as a potent target for chemotherapy due to synergistic effects with current drug regimens. Here, we describe the mechanism of action of a series of antimycobacterial indole-4-carboxamides and the resulting mechanism of resistance Mtb deploys to subvert their cidal effect. We found the series of indole-4-carboxamides to be prodrugs of an antimetabolite, 4-aminoindole, which becomes metabolically incorporated by tryptophan synthase to form the cytotoxic 4-aminotryptophan. Mtb acquired resistance by deploying three discrete mechanisms (1) decrease in drug metabolism, (2) increased biosynthetic flux of tryptophan, and (3) *in situ* enzymatic attenuation of tryptophan synthase. This third method, we propose to be a bona fide resistance mechanism in mycobacteria. Ultimately, these results further demonstrate the complexity involved in Mtb modulation of tryptophan biosynthesis and enhance our understanding of this valuable drug target.**

## STAR★Methods

### Key resources table

**Table:** 

REAGENT or RESOURCE	SOURCE	IDENTIFIER
**Bacterial and virus strains**		

*M. tuberculosis* H37Rv	Lab stock	N/A
*M. bovis* BCG	Lab stock	N/A
*M. smegmatis* mc^2^155	Lab stock	N/A
*E. coli* NEB5α	New England Biolabs	Cat# C2987H
*E. coli* BL21(DE3)	New England Biolabs	Cat# C2527H

**Chemicals, peptides, and recombinant proteins**		

Middlebrook 7H9 broth base (Difco)	FisherScientific	Cat# DF0713-17-9
Middlebrook 7H11 agar base	Millipore Sigma	Cat# M0428-500G
Bovine Serum Albumin Fraction V	Millipore Sigma	Cat# 810535
Gibco Bacto Casitone	FisherScientific	Cat# DF0259-17-9
Tyloxapol	Sigma-Aldrich	Cat# T0307-10G
Tween 80	Sigma-Aldrich	Cat# P7949-500ML
DMEM growth medium	ThermoFisher	Cat# 11965118
Fetal Bovine Serum (FBS)	ThermoFisher	Cat# A4766801
Isoniazid	Sigma-Aldrich	Cat# I3377-5G
Ethionamide	Sigma-Aldrich	Cat# 1261004-200MG
Compounds C1 – C21	This manuscript	N/A
4-aminoindole	Sigma-Aldrich	Cat# 525022-500MG
L-Tryptophan	Sigma-Aldrich	Cat# 93659-10G
L-Serine	Sigma-Aldrich	Cat# 84959-25G
CdRP	This manuscript	N/A
IGP	This manuscript	N/A
4-amino-L-Tryptophan	This manuscript	N/A
5-amino-L-Tryptophan	Sigma-Aldrich	Cat# AC5H30533E13-1G
TrpE WT	This manuscript	N/A
TrpE H170R	This manuscript	N/A
TrpAB WT	This manuscript	N/A
TrpAB βA168V	This manuscript	N/A
TrpAB βD261G	This manuscript	N/A
*E. coli* TrpCF	This manuscript	N/A
GAPDH	Millipore Sigma	Cat# G2267-1KU

**Critical commercial assays**		

CellTiter-Glo Luminescent Kit	Promega	Cat# G7573
BCA Protein Assay Kit	Pierce	Cat# 23227

**Experimental models: cell lines**		

J774A.1	ATCC	ATCC TIB-67

**Experimental models: organisms/strains**		

*M. tuberculosis* H37Rv + pMV306SNKdi-Psmyc-AmiC	This manuscript	N/A
*M. tuberculosis* H37Rv *rpsL*	This manuscript	N/A
*M. tuberculosis* H37Rv *trpB A168V*	This manuscript	N/A
*M. tuberculosis* H37Rv *trpB A134V*	This manuscript	N/A
*M. tuberculosis* H37Rv *trpE H170R*	This manuscript	N/A

**Oligonucleotides**		

Oligonucelotides are listed in Table S5		

**Software and algorithms**		

GraphPad Prism	GraphPad	https://www.graphpad.com/scientific-software/prism/
SeqBuilder Pro	DNAStar	https://www.dnastar.com/software/
PyMol 2.2.3	Schrodinger, LLC	https://pymol.org/2/
ChemDraw	PerkinElmer	https://www.perkinelmer.com/category/chemdraw

### Resource availability

#### Lead contact

Further information and requests for resources and reagents should be directed to and will be fulfilled by the Lead Contact, Clifton E Barry, III (cbarry@niaid.nih.gov)

#### Materials availability

Plasmids generated in this study may be obtained by request to the Lead Contact, recombinant strains of *Mycobacterium tuberculosis* are also available to qualified investigators who possess appropriate safety permits. All other unique/stable reagents generated in this study are available from the lead contact without restriction.

#### Data and code availability

The published article includes all datasets generated during this study.

This study did not generate any unique code.

### Experimental model and subject details

#### Bacterial strains and culture conditions

*M. tuberculosis* and *M. bovis* were obtained from laboratory stocks. *M. tuberculosis* H37Rv or mutants thereof were used for all experiments. Liquid media used most often is 7H9/Gcas (4.7 g/L Middlebrook 7H9 broth base, 4 g/L glucose, 0.8 g/L NaCl, 0.3 g/L Casitone, and 0.05% Tyloxapol), referred to as BSA-free media in the main text. Some MIC measurements were also done in 7H9/ADC (4.7 g/L 7H9 broth base, 5 g/L BSA fraction V, 2 g/L glucose, 0.81 g/L NaCl, 0.02% glycerol, and 0.05% tween 80) or GAST-Fe medium (0.3 g Bacto Casitone (Difco), 4.0 g K_2_HPO_4_, 2.0 g citric acid, 1.0 g L-alanine, 1.2 g MgCl_2_ 6H_2_O, 0.6 g K_2_SO_4_, 2.0 g NH_4_Cl, 1.80 ml 10 NaOH, 10.0 ml glycerol, 0.05% Tween 80 and 0.05 g ferric ammonium citrate adjusted to pH 6.6). Solid medium used where appropriate was Middlebrook 7H11 agar supplemented with 5 g/L dextrose, 2 g/L NaCl, 0.5% glycerol, and 0.06% oleic acid (collectively called 7H11/OADC).

#### Primers and plasmids

All primers used in this study were obtained from Eurofins in their dry and desalted form and reconstituted (as received) in 1X TE buffer to make 100 μM stocks and further diluted in mQ H_2_O to 10 μM. The 10 μM solutions were used for all cloning protocols. All cloning protocols and plasmid isolations were done using commercially available kits from Zymo Research Company.

### Method details

#### Minimum inhibitory concentration

The antimicrobial susceptibility testing against *M. tuberculosis* and *M. bovis* were performed in 7H9/ADC, while those for the resistant mutants (and the corresponding WT control) were done in 7H9/Gcas. Bacteria were grown in the corresponding media up to an OD_600_ of 0.2 to 0.4. A two-fold serial dilution series (50 μL) of the test compounds was placed in each well of a sterile 96-well round bottom plate, and then 50 μL of the bacterial suspension diluted to OD_600_ of 0.0002 was added. The plates were incubated at 37°C for 2 weeks prior to visual determination of the MIC. The MIC is defined here as the drug concentration that completely inhibited growth of cells. MICs measured in the presence of supplementation were done by supplementing the growth medium with 1 mM (or 70 μM) of the corresponding metabolite.

#### Macrophage infection and rescue studies

For the *ex-vivo* efficacy testing, J774.1 cells (2x10^5^ cells/well) were seeded in flat-bottom 24-well plates (Corning Inc.) in DMEM GlutaMAX supplemented with 10% FBS, 20 mM HEPES, and 0.5 mM sodium pyruvate (hereafter abbreviated as DMEM/FBS). The cells were infected with *M. tuberculosis* H37Rv (1x10^6^ cells/well, MOI 1:5) for 24 hrs, followed by removal of the medium and washing twice with Dulbecco PBS. Infected cells were exposed to test compounds at the specified concentrations in fresh growth medium (1 mL/well). Cells were incubated at 37°C, 95% humidity, 5% CO_2_ incubator for 7 days. The medium was changed every 2 days and the infected cells were treated with a fresh batch of drugs each time. After 7 days of incubation, the medium was removed and the cells were washed with PBS twice. Then, 500 μL of 0.1% SDS was added to each well to lyse the J774 cells. After 5 mins, the lysate was thoroughly mixed and diluted in 7H9/ADC and appropriate dilutions were plated in duplicate on 7H11/OADC plates to calculate the most probable number of bacteria. Colonies were counted manually in each plate following 6-8 weeks of incubation at 37°C.

#### J774.1 cytotoxicity

The *in vitro* cytotoxicity of the indole-4-carboxamides were measured in DMED supplemented with 10% FBS. 1x10^4^ J774.1 cells were seeded onto each well of a sterile opaque 96-well plate and left to attach overnight. The next day, the media was aspirated and replaced with a two-fold serial dilution of compounds in the same media. The cells were treated for 24 hrs prior to viability determination using CellTiter-Glo (Promega) according to the manufacturer instructions.

#### Resistant mutant isolation

To raise resistant mutants against compound C1, 10^7^, 10^8^, and 10^9^ cells of *M. tuberculosis* H37Rv were plated on 7H11/OADC plates containing 5x the *in vitro* MIC of C1. Drug-free plates were used to enumerate bacterial load. The plates were incubated at 37°C for 4-6 weeks until colonies grew to an appreciable size. The colonies were picked and inoculated into drug-free 7H9/Gcas medium and the MIC were tested (according to the above protocol) to confirm true resistance against C1. The genomic DNA of the mutants were isolated using the CTAB method (Park et al., 2017) and were sequenced and analyzed as previously described (Ioerger et al., 2010).

#### Cloning of AmiC into pMV306

The mycobacterial shuttle vector pMV361 (Stover et al., 1991) was derivatized in house to generate a non-integrative overexpression vector. The backbone of plasmid pMV361 was amplified using primers 1001 (F) and 1002 (R) (see Table S5), digested with NcoI and reannealed, in order to introduce an NcoI restriction site at the translational start site and simplify future cloning steps. The *L5 integrase* was removed using primers 1003 (F) and 1004 (R) with terminal BamHI restriction sites. Furthermore, the *hsp60* promoter (Stover et al., 1991) was exchanged with Psmyc from pML1357 (Huff et al., 2010) exploiting the XbaI and NcoI restriction sites using primers 1005 (F) and 1006 (R). The new plasmid was named pL5-Psmyc. The *amiC* gene was amplified from *M. tuberculosis* H37Rv genomic DNA using primers 1007 (F) and 1008 (R) and the Q5 Hot-start high-fidelity 2X Master Mix (New England Biolabs). The amplicon and pL5-Psmyc was double digested with NcoI and ClaI; the vector was also treated with alkaline phosphatase (CIP, New England Biolabs) for 30 mins. The DNA fragments were gel purified and the *amiC* fragment was ligated to the vector backbone overnight at room temperature using T4 DNA ligase (New England Biolabs). The ligation reaction was transformed into *E. coli* NEB5α competent cells and the transformants were plated on LB agar containing 50 μg/mL kanamycin. Positive transformants were grown and the plasmid was extracted and confirmed by Sanger sequencing (Eurofins).

#### Overexpression of AmiC in M. tuberculosis H37Rv

Electrocompetent *M. tuberculosis* H37Rv (400 μL) were transferred into a 2mm-gap electroporation cuvette and mixed with 1 μg of pL5-P_smyc_-AmiC. The cells were electroporated using a Bio-Rad GenePulser II electroporator set at the following paramters: 2.5 kV, 1000 Ω, 25 μF. Immediately after electroporation, 1 mL of 7H9/ADC was added to the cells and the mixture was transferred into 10 mL of sterile 7H9/ADC and the cells were allowed to recover for 24 hrs at 37°C. The cells were diluted and plated into 7H11/OADC plates containing 25 μg/mL kanamycin and incubated at 37°C for 4-6 weeks. Electroporation using an empty vector was used as control. The resulting colonies were grown in 7H9/GCas containing 25 μg/mL kanamycin and the MICs were measured using the protocol described above.

#### Compound metabolism in H37Rv

To determine the degree of drug metabolism in live *M. tubeculosis* and the C1^R^ mutants, an LC-MS-based protocol was employed. The cells were grown in 7H9/Gcas to an OD_600_ of 1.0, washed twice with PBS, and resuspended in PBS at 1/3 the original culture volume to afford a suspension at OD_600_ of 3.0. Then, 1 mL of the cell suspension was added to enough compound in DMSO to create a final compound concentration at 10X the *in vitro* MIC. A 200 μL aliquot was removed for the 0 hr time point (immediately after addition of the cells to the drug) and mixed with an equal volume of acetonitrile (ACN) and kept at 4°C. After 24 hrs, another 200 μL aliquot was removed and mixed with ACN. The cells in these aliquots were pelleted and the supernatant was filtered through a 0.2 μm centrifuge filter prior to taking the samples out of the BSL3. A 20 μL aliquot was injected directly into the LC-MS. LC gradient used to separate the metabolites was 5-95% ACN over 20 mins. Chromatograms obtained at 270 nm were used to determine % compound remaining after 24 hrs, and were normalized relative to the 0 hr time point.

#### Determination of 4-aTrp and L-Trp levels following 4-AI treatment

*M. tuberculosis* (100 mL) was grown up in 7H9/ADC to an OD600 of 0.8. Cells were harvested, washed twice with 20 mL PBS and resuspended in 5.6 mL modified Sauton’s medium. The Sauton’s medium consisted of magnesium sulfate heptahydrate (0.5 g/L), potassium dihydrogen phosphate (0.5 g/L), potassium sulfate (725 mg/L), citric acid (2 g/L), ferric ammonium citrate (50 mg/L), L-asparagine (4 g/L), zinc sulfate (0.1 mg/L), glycerol (20 g/L), with pH adjusted to 6.5 with sodium hydroxide (10 M). Half of the cell suspension was treated with 100 mM of 4-aminoindole and the other half with an equivalent amount of DMSO. The samples were divided into 600 mL aliquots and incubated at 37°C in a shaking incubator with 600 mL acetonitrile added at the indicated time points. Samples were stored at -80°C prior to further processing. In a separate experiment, *M. tuberculosis* (700 mL) was grown up in 7H9/Gcas to an OD600 of 0.5. Cells were harvested, washed and treated with 10 mM and 100 mM of 4-aminoindole as above and quenched with 600 mL acetonitrile prior to storage at -80°C.

For tryptophan and 4-aminotryptophan concentration determination (Widner et al., 1997), a stock solution of 3-nitrotyrosine as internal standard, (IS) in 1% heptafluorobutyric acid (HFBA) was made and 20 μL IS solution was added to 200μL sample /acetonitrile mix. Samples were centrifuged to remove precipitant. Concentrations were determined by an Agilent 1290 Infinity HPLC with an Agilent 6460C triple quadrupole mass selective detector. Separation was achieved on an Agilent Eclipse Plus C18 1.8 μm column (dimensions 2.0 x 50 mm) with mobile phase A being aqueous 0.1% HFBA and B being acetonitrile with 0.1% HFBA. 0.8 mL/min flow rate was applied with 7% B starting gradient held 0.25 min, then ramped to 95% B over 4.75 min. Detection was achieved in multiple reaction monitoring mode (MRM) using electrospray ionization with capillary voltage of 2000V and nitrogen gas in collision cell. Tandem mass ions for each precursor M+H^+^ adduct were detected with the product ions m/z 157.1 (CEV 20), 188 (CEV 7), and 181.1 (CEV 10) for 4-aminotryptophan, tryptophan, and IS respectively (CEV = collision energy voltage in volts).

#### Mycobacterial recombineering

To replace the native copies of *trpB* and *trpE* with the mutant alleles, a previously described single-stranded DNA-mediated recombineering protocol was employed (Murphy et al., 2015). 70-mer oligonucleotides were designed to introduce the specific mutations in H37Rv (primers 1009 – 1012 in Table S5). *M. tuberculosis* carrying pKM402 plasmid was grown to OD_600_ of ~ 1.0 in 7H9/ADC. The culture was subcultured to an OD_600_ of 0.1 in 30 mL 7H9/ADC containing 20 μg/mL kanamycin and the cells were allowed to grow to an OD_600_ of ~0.8. RecT expression was induced by addition of anhydrotetracycline (aTc) to a final concentration of 500 ng/mL and allowing the cells incubate overnight at 37°C. Then, 3 mL of a 2M sterile glycine solution was added and the cells were incubated for an additional 16 hrs at 37°C. The cells were collected and washed twice with 10% glycerol and finally resuspended in 3 mL of sterile 10% glycerol. 400 μL of the electrocompetent and recombinogenic *M. tuberculosis* were transferred to a 2mm-gap electroporation cuvette, into which 500 ng of oligos were added. The cells were electroporated using a Bio-Rad Gene Pulser II set at the following parameters: 2.5 kV, 1000 Ω, 25 μF. The cells were removed from the electroporation cuvette and added to 10 mL of 7H9/ADC and allowed to recover at 37°C for 3 days. Then the cells were subcultured 1:100 into 7H9/ADC containing 20 μg/mL kanamycin and allowed to grow to an OD_600_ of ~1.0. The cells were diluted and finally plated on 7H11/OADC plates containing 20 μg/mL streptomycin and incubated at 37°C for 4-6 weeks. Several clones for each recombineering target were selected and genomic DNA was extracted using the CTAB method. The clones were screened for the desired mutations via PCR using primers 1013 – 1018 (see Table S5) followed by Sanger sequencing of the PCR products.

#### Resazurin broth microdilution assay

The resistance of the recombineering and WT strain against NMMV03 compounds were measured using the Alamar Blue Assay. The bacterial strains were grown in 7H9/GCas and the susceptibility testing were done according to the MIC protocol described above. After 7 days of drug treatment, 10 μL of Alamar Blue (ThermoFisher) was added to each well and the plates were returned to the 37°C incubator for 24 hrs. The fluorescence (λ_ex_ = 530 nm, λ_em_ = 590 nm) was read using a ClarioStar multiwell plate reader. The fluorescence of the treated wells were normalized to that of the corresponding untreated well and presented as % cell viability.

#### Cloning, expression and purification of Mtb TrpE

The *M. tuberculosis* H37Rv *trpE* gene was amplified from a genomic DNA template using the Q5 Hot Start High-Fidelity 2X Master Mix (New England Biolabs) and primers 1019 (F) and 1020 (R). The *trpE* amplicon was cloned between the NdeI and HindIII sites of pET28a, resulting in an N-terminal His_6_ tag. The PCR product and pET28a were separately digested with NdeI and HindIII and ligated overnight at room temperature using T4 DNA ligase. The ligation mixture was directly transformed into *E. coli* NEB5α competent cells and the cells were plated onto LB agar + 50 μg/mL kanamycin. The plasmid was extracted and analyzed by Sanger sequencing. To introduce the H170R, the Q5 site-directed mutagenesis kit (New England Biolabs) was used using the pET28a-TrpE construct as template. Primers 1021 (F) and 1022 (R) were used and the resulting Q5 product was transformed into competent NEB5α cells. The H170R mutation was confirmed by Sanger sequencing. The pET28a-TrpE WT and H170R plasmids carrying the correct sequence were then transformed into competent *E. coli* BL21(DE3) and the transformants were plated onto LB agar plates containing 50 μg/mL kanamycin. Positive transformants were inoculated into 20 mL LB + 50 μg/mL kanamycin and grown overnight. The entire overnight culture was transferred into 2 L of LB containing 50 μg/mL kanamycin and allowed to grow until OD_600_ of ~0.6. Protein expression was induced by addition of IPTG to a final concentration of 1 mM. The cells were incubated for an additional 3 hrs at 37°C prior to harvesting by centrifugation at 4000x*g* for 10 mins. The cells were resuspended in 30 mL of lysis buffer (50 mM Tris-HCl, pH 8.0; 500 mM NaCl, 5 mM imidazole, and 1X protease inhibitor cocktail (tablets from Roche)) and disrupted via sonication (cooling on ice for 1 min every 30 sec sonic bursts). The lysate was centrifuged at 45000x*g* for 1 hour and the supernatant was loaded onto a Ni-NTA column equilibrated with 25 mL of column buffer (50 mM Tris-HCl, pH 8.0, 500 mM NaCl, 10 mM EDTA, 5 mM imidazole). The column was washed with 30 mL of column buffer with increasing imidazole concentrations (5 mM, 10 mM, 20 mM, and 40 mM imidazole). The protein was eluted using 10 mL of column buffer containing 300 mM imidazole and collected in 1 mL fractions. The fractions containing purified TrpE (as seen from a Coomassie-stained polyacrylamide gel) were pooled together and concentrated to ~2.5 mL. The protein was buffer exchanged using a PD-10 (Sigma-Aldrich) column equilibrated using 30 mL of storage buffer (20 mM Tris-HCl, 100 mM NaCl, 5 mM MgCl_2_, and 5% glycerol) and eluted from the PD-10 column using 3 mL of storage buffer. The protein was split into 300 μL aliquots and flash frozen using a dry ice/isopropanol bath and stored at -80°C until use. The concentration of the protein preparations was established using the Pierce BCA Assay kit (ThermoFisher) using BSA as a standard, and the concentrations were converted to μM using the calculated molecular weight of TrpE WT and H170R.

#### Mtb TrpE enzyme assay and inhibition studies

The TrpE enzyme kinetics were studied by direct detection of the fluorescent product, anthranilate at λ_ex_ = 320 nm and λ_em_ = 460 nm using a ClarioStar multiwell plate reader. The total volume was 200 μL in all cases and the assay buffer used contained 20 mM Tris-HCl pH 8.0, 100 mM NH_4_Cl, 10 mM MgCl_2_, 100 mM EDTA. The Michaelis-Menten curves were obtained by incubating the enzyme with increasing concentrations of chorismate (dissolved in the assay buffer) where the reaction was started by addition of 0.67 μM of TrpE WT or the H170R mutant. For the inhibition studies, a fixed 50 mM chorismate concentration was used, and increasing concentration of the drugs or L-Trp was added. The fluorescence of anthranilate was converted into concentration values using a standard calibration curve prepared in the same buffer used for kinetic studies. The k_cat_, and IC_50_ values were all calculated using nonlinear curve fitting in GraphPad Prism 8.

#### Cloning and site-directed mutagenesis of Mtb TrpAB

The TrpAB enzyme complex were co-expressed using the pRSFDuet-1 vector tagging TrpB with an N-terminal Hi_6_ epitope in MCS1 and cloning TrpA in MCS2. *trpB* was amplified from H37Rv genomic DNA using primers 1023 (F) and 1024 (R) and Q5 Hot-start high-fidelity 2X master mix (New England Biolabs). The amplicon and pRSFDuet-1 were digested with HindIII for 1 hr; the vector was also treated with alkaline phosphatase (CIP, New England Biolabs) for 30 mins. The DNA fragments were gel purified and ligated using T4 DNA ligase (New England Biolabs) overnight at room temperature. The ligation mixture was transformed into competent *E. coli* NEB5α cells, the plasmid was extracted, and the sequence was verified using Sanger sequencing (Eurofins). Then, *trpA* was cloned into pRSFDuet-TrpB^MCS1^ in a similar manner between the NdeI and XhoI sites. The resulting pRSFDuet-TrpB^MCS1^-TrpA^MCS2^ construct was fully sequence to verify the sequence of both coding regions and the overall integrity of the plasmid. Initial expression studies in BL21(DE3) cells using this construct showed significant difference in the expression level between TrpB and TrpA (~5:1 TrpB:TrpA).

To increase expression of TrpA, we prepared another construct and co-introduced the two plasmids into the expression vector. The TrpA supplemental expression plasmid was also made using the pRSFDuet-1 vector. Briefly, the native Kan^R^ marker of pRSFDuet-1 was replaced by amplifying the Amp^R^ cassette from pUC19 using primers 1027 (F) and 1028 (R) and cloning the amplicon between the AgeI and SphI restriction sites of pRSFDuet-1. The resulting pRSFDuet^AmpR^ was used as a backbone for TrpA. TrpA was amplified from H37Rv genomic DNA using primers 1029 (F) and 1030 (R) and Q5 Hot-start high-fidelity 2X master mix (New England Biolabs). The amplicon and pRSFDuet^AmpR^ was double digested with NcoI and HindIII; the vector was also treated with alkaline phsphatase (CIP, New England Biolabs). The DNA digests were gel purified and ligated using T4 DNA ligase (New England Biolabs) overnight at room temperature. The ligation was transformed into competent *E. coli* NEB5α cells and plated on LB agar containing 100 μg/mL Ampicillin. The positive transformants were grown, the resulting plasmid (pRSFDuet^AmpR^-TrpA^MCS1^) extracted and analyzed by Sanger sequencing (Eurofins).

To introduce the βA168V and the βD261G mutations, the Q5 site-directed mutagenesis kit (New England Biolabs) was used using the pRSFDuet-TrpB^MCS1^-TrpA^MCS2^ construct as template. Primers pairs 1031 (F) – 1032 (R) and 1033 (F) – 1034 (R) were used and the resulting Q5 product was transformed into competent NEB5α cells. Introduction of the mutations were confirmed by Sanger sequencing (Eurofins).

#### Overexpression and purification of Mtb TrpAB

Competent *E. coli* BL21(DE3) were co-transformed with pRSFDuet-TrpB^MCS1^-TrpA^MCS2^ and pRSFDuet^AmpR^-TrpA^MCS1^ and grown in LB + 50 μg/mL kanamycin and + 100 μg/mL ampicillin. The transformed BL21(DE3) cells were grown in LB media at 37°C until an OD_600_ of 0.6-0.7. Expression was induced with IPTG (1 mM final concentration) and incubated for 3 hours at 37°C. Cells were pelleted by centrifugation at 4000rpm for 20 minutes and then resuspended in lysis buffer (50 mM HEPES pH 8.0, 150 mM KCl, 5% glycerol, 10 mM Imidazole, 1 mM PLP, 1 mM TCEP, and 1X Protease Inhibitor Cocktail Tablet (Roche)). After sonication, the lysed cells were pelleted by centrifugation at 15,000 rpm for 60 minutes. The supernatant was loaded onto a Ni-NTA column, and then washed with a wash buffer (50mM HEPES pH 8.0, 100mM KCl, 5% glycerol, 1 mM PLP, 1mM TCEP, and a 10-40mM gradient of Imidazole). After washing, the loaded protein was eluted with an elution buffer (Same as wash buffer but with 300 mM Imidazole). The eluted protein was dialyzed overnight against a storage buffer (20mM HEPES pH 8.0, 100 mM KCl, 5% glycerol, 40 μM PLP, 0.5 mM TCEP). TrpAB were identified using SDS-PAGE gel, aliquoted, and stored at -80°C until use. The concentration of the complex was measured spectrophotometrically using the Pierce BCA Assay kit (ThermoFisher) and the accompanying BSA standards. The concentrations calculated from the calibration curve were converted to μM units using the molecular weight of the complex. Due to the higher expression of TrpB in comparison to TrpA (~2:1 TrpB:TrpA), concentration of the TrpAB complex was adjusted to the percent TrpA present, assuming any extra TrpB was inactive enzyme.

#### Preparation of Indole-3-glyceraldehyde phosphate (IGP)

To test the α-reaction of TrpAB, isolation and purification of the substrate indole-3-glycerol phosphate (IGP) was necessary. IGP is not available commercially and no chemical synthesis method has been published, so we developed a method to enzymatically synthesize IGP. We used *E. coli* indole-3-glycerol phosphate synthase (TrpCF) to convert 1-(o-carboxyphenylamino)-deoxyribulose 5-phosphate (CdRP) to IGP via an irreversible ring closure reaction spurred by decarboxylation and dehydration steps. The *trpCF* gene was amplified from *E. coli* MG1655 genomic DNA using primers 1035 (F) and 1036 (R) and Q5 Hot-start high-fidelity 2X master mix (New England Biolabs). The amplicon and pET28a was double digested with NdeI and HindIII; the vector was also treated with alkaline phsphatase (CIP, New England Biolabs). The DNA digests were gel purified and ligated using T4 DNA ligase (New England Biolabs) overnight at room temperature. The ligation mixture was transformed into competent *E. coli* NEB5α, the pET28a-(Ec)TrpCF plasmid extracted and analyzed via Sanger sequencing (Eurofins). The plasmid carrying the correct sequence was transformed into *E. coli* BL21(DE3) cells grown in the presence of 50 μg/mL kanamycin. An overnight culture of BL21 expressing TrpCF was transferred to 2 L of LB + 50 μg/mL kanamycin and allowed to grow to an OD_600_ of 0.6. Protein expression was induced by addition of IPTG to a final concentration of 500 μM and incubation for an additional 3 hrs at 37°C. The cells were harvested by centrifugation at 4000x*g* for 10 mins. The cells were resuspended in 30 mL of lysis buffer (50 mM Tris-HCl, pH 8.0; 500 mM NaCl, 5 mM imidazole, and 1X protease inhibitor cocktail (tablets from Roche)) and disrupted via sonication (cooling on ice for 1 min every 30 sec sonic bursts). The lysate was centrifuged at 45000x*g* for 1 hour and the supernatant was loaded onto a Ni-NTA column equilibrated with 25 mL of column buffer (20 mM Tris-HCl, pH 8.0, 200 mM NaCl, 5 mM imidazole). The column was washed with 30 mL of column buffer with increasing imidazole concentrations (5 mM, 10 mM, 20 mM, and 40 mM imidazole). The protein was eluted using 10 mL of column buffer containing 300 mM imidazole and collected in 1 mL fractions. The fractions containing purified TrpCF (as seen from a Coomassie-stained polyacrylamide gel) were pooled together and concentrated to ~2.5 mL. The protein was buffer exchanged using a PD-10 (Sigma-Aldrich) column equilibrated using 30 mL of storage buffer (20 mM Tris-HCl, and 5% glycerol) and eluted from the PD-10 column using 3 mL of storage buffer. The protein were split into 300 μL aliquots and flash frozen using a dry ice/isopropanol bath and stored at -80°C until use. The protein concentration was measured spectrophotometrically using the Pierce BCA Assay kit (ThermoFisher) and the accompanying BSA standards.

To generate CdRP, we followed chemical synthesis protocols previously reported (Czekster et al., 2009). After desiccation, CdRP powder was protected from light and stored at -20C. In preparation for enzymatic conversion, 10mM CdRP was dissolved in 1.0 M Tris-HCl, pH 7.5. To a reaction volume of 2 mL, the 10 mM CdRP was incubated with ~100ug of *E. coli* TrpCF overnight at 37°C. After incubation, to separate out the enzyme from IGP, the solution was loaded onto a 10,000MW filter and centrifuged. The resulting elute was aliquoted and stored at -20C. Conversion of CdRP to IGP was confirmed via LC-MS. Because no CdRP could be detected via LC-MS after overnight incubation, the concentration of resulting IGP was calculated by incubating IGP overnight with excess TrpAB and quantifying the amount of L-Trp present with LC-MS-based L-Trp standard curve.

#### Kinetic characterization of recombinant Mtb TrpAB

The TrpAB kinetics was monitored with an absorbance-based assay using a ClarioStar multiwell plate reader. The activity of the α-subunit was coupled to the activity of glyceraldehyde-3-phosphate dehydrogenase (GAPDH). 100 nM TrpAB was prepared in the TrpAB storage buffer with 2.5 mM NAD^+^, 30 mM sodium arsenate, 1 μg GAPDH, and increasing amounts of either IGP or L-Serine. The K_M_ and k_cat_ values for the α-reaction were calculated using the rates obtained from varying IGP concentrations. Because the activity of the TrpAB subunits is tightly coupled, such that no TrpA product will be produced without the binding of L-Ser to TrpB, we utilized as an analog, the formation of G3P from TrpA (under varying concentrations of L-Ser) to indirectly monitor the progress of the β-reaction. Therefore, K_M_ and k_cat_ values for the β-reaction were calculated using the rates obtained from increasing concentrations of L-Ser. The enzyme mix was aliquoted into the wells of clear, flat bottom 96-well plate. The reaction was initiated with the addition of substrate and monitored by following the NADH absorbance at 340nm (ε_NADH_ = 6300 M^-1^ cm^-1^).

To monitor the β-reaction exclusively (Indole + L-Ser → L-Trp) a spectrophotometric monitoring was employed (Shen et al., 2009; Xu and Abeles, 1993) using a ClarioStar multiwell plate reader. 50 nM of TrpAB WT or 200 nM of TrpAB mutants (the mutants exhibited significant attenuation in enzymatic activity that we could only reproducibly assay the kinetics at higher enzyme concentrations) were prepared in TrpAB storage buffer containing 40 μM PLP and 20 mM L-Ser. To the enzyme mix was added increasing concentrations of indole (stock solution prepared at 100 mM in MeOH and diluted to the appropriate concentrations in the TrpAB storage buffer) and the progress of reaction was monitored at 290 nm. The absorbance values were converted to rates using the difference in absorption between indole and L-Trp at 290 nm (Δε = 1850 M^-1^ cm^-1^) (Xu and Abeles, 1993). All kinetic measurements were done at 25°C.

#### Mtb TrpAB inhibition studies

Inhibition of the α-reaction was monitored by coupling the activity of the α-subunit to that of GAPDH. The mixtures (total volume 200 μL) were prepared in TrpAB storage buffer and included 100 nM TrpAB, 50 μM IGP, 20 mM L-Ser, 2.5 mM NAD^+^, 30 mM sodium arsenate, 1 μg GAPDH, and increasing concentrations of inhibitors. The reaction was initiated by addition of the enzyme and progress was monitored by following the NADH absorbance at 340nm (ε_NADH_ = 6300 M^-1^ cm^-1^). Inhibition of the β-reaction was monitored at 290 nm and performed as described above. The mixtures (total volume 200 μL) were prepared in TrpAB storage buffer and included 100 nM TrpAB, 0.1 mM Indole, and 3 mM L-Ser (K_M_^L-Ser^ of TrpAB β-half reaction was calculated to be 2.9 ± 0.3 mM in a separate determination, data not shown) in the presence of increasing inhibitor concentrations. The reaction was initiated by addition of the enzyme and the progress was monitored by recording the absorbance at 290 nm. All inhibition studies were done at 25°C.

#### Activity of Mtb TrpAB β-reaction using 4-AI as substrate

A LC-MS-based assay was developed to detect and quantify the conversion of 4-AI to 4-aTrp. 50 nM TrpAB WT and mutant TrpAB were prepared in TrpAB buffer with 30 mM L-Serine. The reaction was initiated by the addition of increasing amounts of 4-aminoindole. After 5 minutes of incubation, the reaction was quenched with an equal volume of 0.1% formic acid in methanol followed by centrifugation. 2 μL of the collected supernatant was injected into the LC-MS. 4-aminotryptophan was separated and detected on an Agilent 1100 HPLC system with a single quadrupole mass selective detector (G1946) using a Waters Acquity BEH Amide 2.1 mm by 50mm 1.7mm particle column. The mobile phase was aqueous with 0.1% formic acid (channel A) and Acetonitrile with 0.1% formic acid (channel B). Gradient of 95% B was initiated and linearly changed to 50% B over 10 minutes at flow rate 0.3 mL/min. Compounds were quantified by diode array detector with absorbance at 220nm. Tandem confirmation was implemented by scanning in positive mode with electrospray ionization (ESI^+^) for the M+H^+^ adduct at 220m/z. Response units obtained from the LC-MS were converted to μM units using a separately determined calibration curve using synthetic 4-aTrp.

Further confirmation of 4-aTrp by TrpAB WT was determined on an Agilent 1290 infinity with triple quadrupole mass selective detector (6460C). Confirmation was determined by retention time (same column / mobile phase) and energy resolved mass spectrometry (erms) profile with CEV 0-70V (E = 0 to 7.9eV) for both enzymatic product and synthetic standard.

#### Intracellular metabolic incorporation of 4-AI into 4-aTrp

The 4-aTrp concentration following 4-AI treatment of Mtb was done exactly the same as the compound metabolism studies described above, except that to quench the metabolism, the aliquots were transferred to an equal volume of 1:1 ACN:MeOH mixture prior to filtering out the cells in the mixture. The 4-aTrp was detected and quantified using the LC-MS equipped with BEH amide column. Concentrations were normalized against the WT run of the first trial.

#### General chemistry information

##### Abbreviations list

AcOH-acetic acid

DCM-dichloromethane

DIPEA-N,N-diisopropylethylamine

DMAP- 4-Dimethylaminopyridine

DMF-N,N-dimethylformamide

DMSO-dimethyl sulfoxide

EDCI- 1-Ethyl-3-(3-dimethylaminopropyl)carbodiimide

EtOAc-ethyl acetate

EtOH-ethanol

FA-formic acid

HATU-1-[Bis(dimethylamino)methylene]-1H-1,2,3-triazolo[4,5-b]pyridinium-3-oxide hexafluorophosphate

HOBt-1-hydroxybenzotriazole

MeCN-acetonitrile

MeOH-methanol

THF-tetrahydrofuan

All reagents including solvents used in the synthetic procedure were purchased from Sigma-Aldrich and used without further purification unless otherwise mentioned. NMR was performed by either a Bruker AVANCE III HD NanoBay 400 MHz, Spectrometer or Bruker Avance DPX 400, Bruker Avance DPX 500 spectrometer, and chemical shifts were measured in ppm relative to specific solvent signal. Routine mass and purity analyses (LRMS) were performed on:

• HP Agilent LC/MS series 1100 system equipped with a reverse phase column (Agilent Poroshell 120 EC-C18, 2.7 μm, 50 X 2.1 mm) and photodiode array detector coupled to an Agilent 1946 DSL quadrupole mass selective detector using electrospray ionization (ESI).
• Bruker MicrOTOF II focus ESI Mass Spectrometer connected in parallel to Dionex Ultimate 3000 RSLC system with diode array detector equipped with a reverse phase column (Waters XBridge C18 column, 2.1 x 50mm, 3.5 μm particle size) using ESI.
• HP Agilent LC/MS series 1100 system equipped with a reverse phase column (Kinetex EVO C18 column, 5 μm, 30 x 2.1 mm) and photodiode array detector coupled to an Agilent G1956A quadrupole mass selective detector using electrospray ionization (ESI)
• HP Agilent LC/MS series 1260 system equipped with a reverse phase column (Kinetex EVO C18 column, 5 μm, 30 x 2.1 mm) and photodiode array detector coupled to an Agilent G6110A quadrupole mass selective detector using electrospray ionization (ESI)
• Shimadzu LC20A system equipped with a reverse phase column (Kinetex EVO C18 column, 5 μm, 30 x 2.1 mm) and photodiode array detector coupled to a Shimadzu LCMS-2020 mass selective detector using electrospray ionization (ESI)
• The compounds were eluted with a gradient of 5 to 95% MeCN/water +0.025 or 0.1% Ammonia, or +0.1% FA or 0.0375% TFA.

The purification of compounds was performed by flash column purification using SiliaFlash® P60 (40–60 μm) or by preparative HPLC using one of the following methods:

• Method A. Varian preparative HPLC system equipped with a reverse phase column (Phenomenex Luna C18, 10 μm, 250 x 21.2 mm) and photodiode array detector.
• Method B: Gilson preparative HPLC system equipped with a reverse phase column (Column: Phenomenex Synergi C18 150∗25∗10 μmum) and a photodiode array detector; Mmobile phase: [water (0.225% FA)-MeCN]; B%: 35%-65%, 10 min)
• Method C: Gilson preparative HPLC system equipped with a reverse phase column Column: PPhenomenex Synergi C18 150∗30 mm∗4 μmum) and a photodiode array detector; Mmobile phase: [water (0.225% FA)-MeCN]; B%: 45%-72%, 12 min)
• Method D: Gilson preparative HPLC system equipped with a reverse phase column Column: Phenomenex Synergi C18 150∗25∗10 μmum) and a photodiode array detector; Mobile phase: [water (0.225% FA)-MeCN]; B%: 20%-50%, 10 min)
• Method E: Gilson preparative HPLC system equipped with a reverse phase column Column: Phenomenex Gemini 150∗25 mm∗10 μmum) and a photodiode array detector; Mobile phase: [water (0.04% NH4OH+10mM NH4HCO3)-MeCN]; B%: 35%-65%, 10 min
• Method F: Gilson preparative HPLC system equipped with a reverse phase column ( Phenomenex Synergi C18 150∗25∗10 μm) and a photodiode array detector; Mobile phase: [water (0.225% FA)-MeCN]; B%: 50-80%, 10 min)
• Method G: Gilson preparative HPLC system equipped with a reverse phase column (Phenomenex column: Gemini 150∗25 5μmu) and a photodiode array detector; mMobile phase: [water (0.05% ammonia hydroxide v/v)-MeCN]; B%: 46%-76%, 12 min
• Method H: Gilson preparative HPLC system equipped with a reverse phase column: Phenomenex Gemini 150∗25mm∗10μmum) and a photodiode array detector; mMobile phase: [water (0.04%NH4OH+10mM NH4HCO3)-MeCN]; B%: 37%-67%, 9 min
• Method I: Gilson preparative HPLC system equipped with a reverse phase column Phenomenex Gemini 150∗25mm∗10uμmm) and a photodiode array detector; mobile phase: [water (0.04%NH4OH+10mM NH4HCO3)-MeCN]; B%: 40%-70%, 9.5 min
• Method J: Gilson preparative HPLC system equipped with a reverse phase column column: Phenomenex Synergi C18 150∗25∗10μmum) and a photodiode array detector; mobile phase: [water (0.225%FA)-MeCN]; B%: 40%-70%, 9min
• Method K: Enamine Instrument: Agilent 1260 Infinity HPLC systems equipped with a reverse phase column DAD and mass-detector Column: (Waters Sunfire C18 OBD Prep Column, 100 A, 5 μm, 19 mm x 100mm with SunFire C18 Prep Guard Cartridge, 100 A, 10 μm, 19 mm x 10 mm) Detection: DAD – DAD1A 215 nm, DAD1B 254 nm. MSD – single quadrupole, AP-ESI Mobile phases: A - Deionized water (100%). B - HPLC-grade MeOH (100%) Gradient: from A-50%: B-50% to A-0%: B-100%.
• Method L: Gilson preparative HPLC system equipped with a reverse phase column column: Phenomenex Synergi C18 150∗25∗10μmum) and a photodiode array detector; Mmobile phase: [water (0.225%FA)-MeCN]; B%: 45%-75%, 9min

All reagents including solvents used in the synthetic procedure were purchased from Sigma-Aldrich and used without further purification unless otherwise mentioned. Synthesized compounds were ≥95% pure (by LCMS and ^1^H NMR) unless otherwise stated.

##### Synthesis of mixture, 4-nitro-L-tryptophan and 6-nitro-L-tryptophan (1)

To a suspension of L-tryptophan (0.2g, 0.97 mmol) in 4 mL AcOH and 1 mL H_2_O was dropwise added 70% HNO_3_ (0.2 mL, 2.95 mmol). The reaction mixture was stirred at room T for about 4 h. After evaporation, it was purified by preparative HPLC system in the condition of 20 mL/min of flow rate with linear gradient over 30 min (solvent A was 0.1% HCOOH in water and B was 0.1% HCOOH in ACN, the detection wavelength was 330 nm). A yellow solid mixture of 4- and 6-nitro-L-tryptophan was obtained (50 mg, 20% yield): 1H NMR (400MHz, DMSO-*d6*) δ 8.34 (d, J = 1.9 Hz, 1H), 7.97−7.93 (m, 1.5H), 7.79−7.76 (m, 1.5H), 7.56 (s, 1H), 7.50 (s, 0.5H), 7.26 (t, J = 8.0 Hz, 0.5H), 4.11−4.03 (m, 1.5H), 3.74 (dd, J = 5.5 and 14.7 Hz, 0.5H), 3.49 (dd, J = 4.6 and 15.2 Hz, 1H), 3.37−3.30 (m, 1.5H); LRMS (ESI) m/z 250 [M + H]+.

##### Synthesis of Boc protected 4-nitro-L-tryptophan (**2**)

To a solution of 1 (50 mg, 0.2 mmol) in 5 mL of MC were added Boc anhydride (0.13g, 0.6 mmol), DMAP (0.07g, 0.6 mmol), and NEt_3_ (0.11 mL, 0.8 mmol). The reaction mixture was stirred overnight at room. The reaction mixture was diluted with EtOH and washed with 0.1N HCl and brine. The organic layer was dried over Na2SO4 and evaporated in vacuo. The crude mixture was purified by flash column chromatography (EtOAc / hexane = 4 / 1 with 0.4% AcOH) to obtain the desired product **2** (20 mg, 22%): ^1^H NMR (400MHz, CDCl_3_) δ 8.56 (d, J = 7.6 Hz, 1H), 7.88 (d, J = 7.9 Hz, 1H), 7.72 (s, 1H), 7.36 (t, J = 7.9 Hz, 1H), 5.14 (d, J = 8.4 Hz, 1H), 4.59 (d, J = 4.4 Hz, 1H), 3.47 (dd, J = 3.2 and 15.1 Hz, 1H), 3.15 (dd, J = 9.3 and 14.7 Hz, 1H), 1.67 (s, 9H), 1.33 (s, 9H); LRMS (ESI) m/z 448 [M - H]-.

##### Synthesis of 4-amino-L-tryptophan (**4**)

To a solution of 1 (20 mg) in 3 mL MeOH was added 5 mg of 10 wt.% Pd/C. The reaction mixture was stirred for about 2 h at room temperature under a H_2_ balloon. After filtration of the reaction mixture through a pad of celite, it was evaporated and used in the next reaction without further purification (**3**). The evaporated residue (**3**) was dissolved in 1 mL of MC and 1 mL of TFA. The reaction mixture was stirred for about 2 h at room T. After evaporation, it was purified by preparative HPLC system in the condition of 20 mL/min of flow rate with A / B = 95 / 5 over 10 min (solvent A was 0.1% HCOOH in water and B was 0.1% HCOOH in ACN, the detection wavelength was 270 nm) to obtain desired product **4** as a TFA salt (13 mg, 87%): ^1^H NMR (400MHz, D_2_O) δ 7.58 (d, J = 8.3 Hz, 1H), 7.43 (s, 1H), 7.25 (t, J = 7.9 Hz, 1H), 7.11 (d, J = 7.4 Hz, 1H), 4.23 (t, J = 5.0 Hz, 1H), 3.57 (dd, J = 5.4 and 16.2 Hz, 1H), 3.46 (dd, J = 4.2 and 16.0 Hz, 1H); ^13^C NMR (100 MHz, D_2_O) δ 162.9, 162.5, 137.9, 127.3, 121.9, 121.8, 121.6, 119.4, 114.7, 113.2, 104.4, 25.8; LRMS (ESI) m/z 220 [M + H]+.

##### N-(1H-indol-4-yl)-2-phenoxyacetamide (**C2**)

**Figure:**
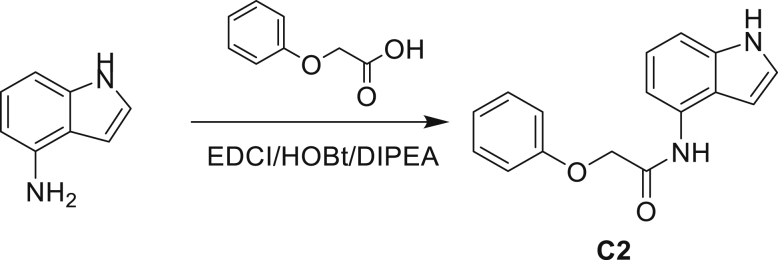

To a solution of 1H-indol-4-amine (104 mg, 0.788mmol) and 2-phenoxyacetic acid (100 mg, 0.657 mmol in DMF (1 mL) was added EDCI (189 mg, 0.985 mmol), HOBt (133 mg, 0.985 mmol) and DIPEA (0.343 mL,1.97 mmol) and the solution was stirred at 25°C for 20 hours. The mixture was poured into an ice-water (5 mL), the resulting precipitate collected by filtration, washed with water (2 mL×3) and dried under vacuum. The crude product was purified by Prep-HPLC (Method B) to give the desired compound **C2** (54.0 mg, 30.5% yield) as a dark solid. ^1^H NMR (400MHz, DMSO-d_6_) δ 1.18 (br s, 1H), 9.76 (s, 1H), 7.54 (br d, J=7.5 Hz, 1H), 7.39 - 7.28 (m, 3H), 7.20 (br d, J=7.9 Hz, 1H), 7.08 - 6.95 (m, 4H), 6.60 (br s, 1H), 4.82 (s, 2H); LRMS (ESI) m/z 267.0 [M + H]+.

##### N-(1H-indol-4-yl)cyclohexanecarboxamide (**C3**)

**Figure:**
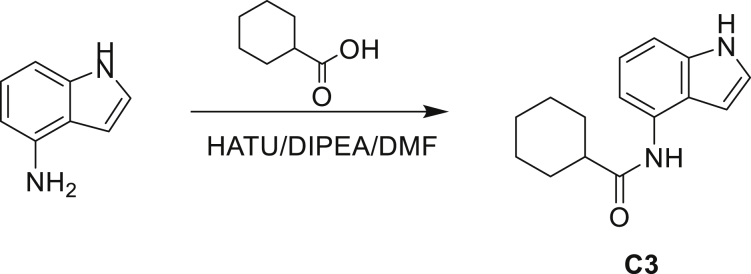

To a solution of cyclohexanecarboxylic acid (100 mg, 0.78 mmol) and 1H-indol-4-amine (123 mg, 0.936 mmol) in DMF (2 mL) was added HATU (593 mg, 1.56 mmol) and DIPEA (504 mg, 3.90 mmol) and the solution was stirred at 25°C for 4 hours. The mixture was poured into an ice-water (5 mL), the resulting precipitate collected by filtration, washed with water (2 mL×3) and dried under vacuum. The crude product was purified by Prep-HPLC (Method B) to give the desired compound **C3** (50.4 mg, 96.4% yield) as a gray solid. ^1^H NMR (400 MHz, DMSO-d_6_): δ 11.07 (br s, 1H), 9.40 (s, 1H), 7.57 (d, J=7.6 Hz, 1H), 7.27 (t, J=2.8 Hz, 1H), 7.12 (d, J=8.2Hz, 1H), 7.04 – 6.95 (m, 1H), 6.70 (br s, 1H), 2.53 (br s, 1H), 1.86 – 1.75 (m, 4H), 1.67 (br d, J=11.4 Hz, 1H), 1.50 – 1.39 (m, 2H), 1.34 – 1.18 (m, 3H); LRMS (ESI) m/z 243.1 [M + H]+

##### N-(1H-indol-4-yl)-5-(2,2,2-trifluoroethoxy)picolinamide (**C4**)

###### Step 1

**Figure:**
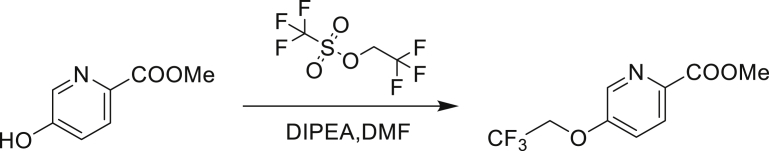

To a mixture of methyl 5-hydroxypicolinate (200 mg, 1.31 mmol) and 2,2,2-trifluoroethyl trifluoromethanesulfonate (454 mg, 1.96 mmol) in MeCN (5 mL) was added cesium carbonate (638 mg, 1.96 mmol) at 0°C and the mixture was stirred at 25°C for 10 hours. The reaction was concentrated *in vacuo* to remove most of the solvent to give a crude residue which was poured into saturated ammonium chloride solution (200 mL) and stirred for 5 min. The pH of the mixture was adjusted to pH=3 with HCl (aq,1M) and the aqueous phase was extracted with EtOAc (30 mL×3). The combined organic phases were washed with brine (20 mL), dried with anhydrous anhydrous sodium sulfate, filtered and concentrated *in vacuo* to give methyl 5-(2,2,2-trifluoroethoxy)picolinate (180 mg, 50.5% yield) as a white solid which was used without further purification. ^**1**^**H NMR** (400MHz, DMSO-d6) δ= 8.56 (d, J=2.8 Hz, 1H), 8.14 (d, J=8.8 Hz, 1H), 7.73 (dd, J=3.2, 8.8 Hz, 1H), 5.05 (q, J=8.8 Hz, 2H), 3.91 (s, 3H); LRMS (ESI) m/z 236.1 [M + H]+

###### Step 2

**Figure:**
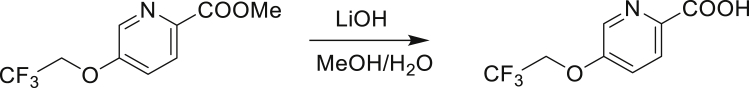

To a mixture of give methyl 5-(2,2,2-trifluoroethoxy)picolinate (180 mg, 0.765 umol) in MeOH (6 mL) was added a solution of lithium hydroxide monohydrate (96.4 mg, 2.30 mmol) in water (2 mL) at 25°C and the mixture was stirred at 25°C for 12 hours. The pH of the mixture was adjusted to pH=5 with AcOH (0.15 mL), stirred for 20 min and extracted with EtOAc (30 mL×3). The combined organic phase was washed with brine (30 mL), dried with anhydrous anhydrous sodium sulfate, filtered and concentrated *in vacuo* to give 5-(2,2,2-trifluoroethoxy)picolinic acid (110 mg, 64.4% yield) as a white solid. LRMS (ESI) m/z 222.0 [M + H]+

###### Step 3

**Figure:**
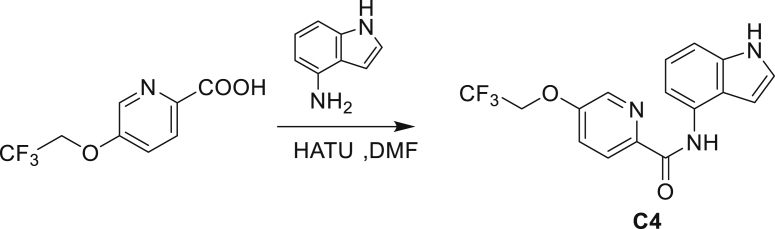

To a mixture of 5-(2,2,2-trifluoroethoxy)picolinic acid (100 mg, 0.452 mmol) and 1H-indol-4-amine (59.8 mg, 0.452 mmol) in DMF (5 mL) was added HATU (343 mg, 0.904 mmol) and DIPEA (292.23 mg, 2.26 mmol) in one portion at 25°C and the mixture was stirred for 10 hours. The mixture was filtered and the filtrate was concentrated *in vacuo* to give a crude residue which was purified by prep-HPLC (Method C) to give the desired compound **C4** (59.5 mg, 39.0% yield) as a white solid. ^1^H NMR (400MHz, DMSO-d_6_) δ = 11.28 (br s, 1H), 10.26 (s, 1H), 8.58 (d, J=2.8 Hz, 1H), 8.21 (d, J=8.7 Hz, 1H), 7.83 - 7.76 (m, 2H), 7.39 (t, J=2.8 Hz, 1H), 7.24 (d, J=8.1 Hz, 1H), 7.11 (t, J=7.9 Hz, 1H), 6.52 (br s, 1H), 5.04 (q, J=8.8 Hz, 2H); LRMS (ESI) m/z 336.1 [M + H]+

##### N-(1H-indol-5-yl)-6-(2,2,2-trifluoroethoxy)nicotinamide (**C6**)

**Figure:**
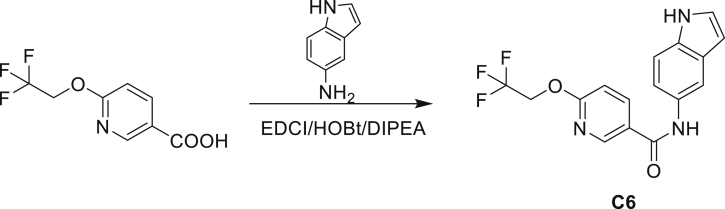

To a solution of 1H-indol-5-amine (35.8 mg, 0.271 mmol) and 6-(2,2,2-trifluoroethoxy)nicotinic acid (50 mg, 0.226 mmol, 1 eq) in DMF (0.5 mL) was added EDCI (65.0 mg, 0.339 mmol), HOBt (45.8 mg, 0.339mmol) and DIPEA (87.6 mg, 0.678mmol and the solution was stirred at 25°C for 20 hours. The mixture was poured into an ice-water (5 mL), the resulting precipitate collected by filtration, washed with water (2 mL×3) and dried under vacuum. The crude product was purified by Prep-HPLC (Method D) to give the desired compound **C6** (20.47 mg, 27.0% yield) was obtained as a white solid. ^1^H NMR (400 MHz, DMSO-d6): δ 11.06 (br s, 1H), 10.17 (s, 1H), 8.82 (d, J=2.2 Hz, 1H), 8.35 (dd, J=2.4, 8.6 Hz, 1H), 7.97 (s, 1H), 7.40 - 7.30 (m, 3H), 7.14 (d, J=8.7 Hz, 1H), 6.45 - 6.39 (m, 1H), 5.11 (q, J=9.0 Hz, 2H); LRMS (ESI) m/z 336.0 [M + H]+.

##### N-(1H-indol-6-yl)-6-(2,2,2-trifluoroethoxy)nicotinamide (**C7**)

**Figure:**
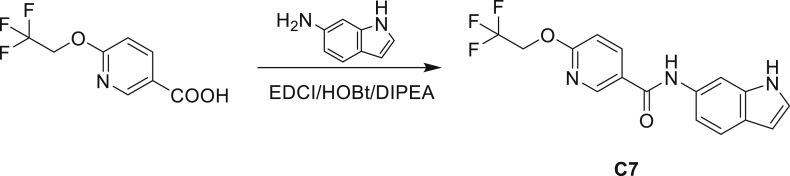

Using a similar method to **C6**, except using 1H-indol-6-amine (35.8 mg, 0.271 mmol) the desired compound **C7** (18.8 mg, 24.8% yield) was obtained as a white solid. ^1^H NMR (400 MHz, DMSO-d_6_): δ 11.08 (br s, 1H), 10.25 (s, 1H), 8.81 (d, J=2.1 Hz, 1H), 8.35 (dd, J=2.4, 8.6 Hz, 1H), 8.05 (s, 1H), 7.49 (d, J=8.6 Hz, 1H), 7.34 - 7.22 (m, 2H), 7.14 (d, J=8.6 Hz, 1H), 6.39 (br s, 1H), 5.11 (q, J=9.0 Hz, 2H); LRMS (ESI) m/z 336.0 [M + H]+.

##### N-(1H-indol-7-yl)-6-(2,2,2-trifluoroethoxy)nicotinamide **(C8)**

**Figure:**
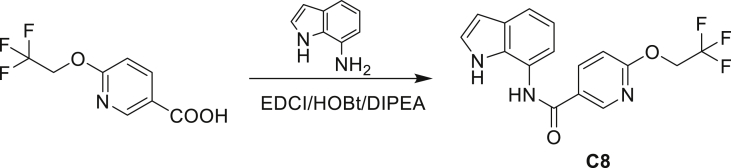

Using a similar method to **C6**, except using 1H-indol-7-amine (35.8 mg, 0.271 mmol), the desired compound **C8** (39.1 mg, 51.6% yield) was obtained as a white solid. ^1^H NMR (400 MHz, DMSO-d_6_): δ 10.92 (br s, 1H), 10.22 (s, 1H), 8.90 (d, J=2.2 Hz, 1H), 8.41 (dd, J=2.4, 8.7 Hz, 1H), 7.50 - 7.27 (m, 3H), 7.17 (d, J=8.7 Hz, 1H), 7.01 (t, J=7.7 Hz, 1H), 6.48 (dd, J=2.0, 2.9 Hz, 1H), 5.13 (q, J=9.0 Hz, 2H); LRMS (ESI) m/z 336.0 [M + H]+.

##### N-(1-methyl-1H-indol-4-yl)-6-(2,2,2-trifluoroethoxy)nicotinamide (**C9**)

**Figure:**
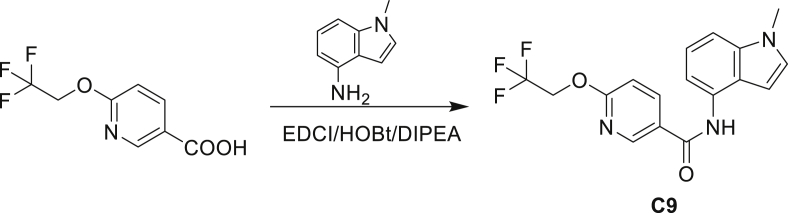

Using a similar method to **C6**, except using 1-methylindol-4-amine (39.6 mg, 0.271 mmol), the desired compound **C9** (30.2 mg, 38.1% yield) was obtained as a white solid. ^1^H NMR (400 MHz, DMSO-d6): δ 10.20 (s, 1H), 8.85 (d, J=2.1 Hz, 1H), 8.37 (dd, J=2.3, 8.7 Hz, 1H), 7.49 - 7.09 (m, 5H), 6.61 (d, J=2.9 Hz, 1H), 5.12 (q, J=9.0 Hz, 2H), 3.81 (s, 3H); LRMS (ESI) m/z 350.0 [M + H]+.

##### N-((6-(2,2,2-trifluoroethoxy)pyridin-3-yl)methyl)-1H-indol-4-amine (**C10**)

###### Step 1

**Figure:**
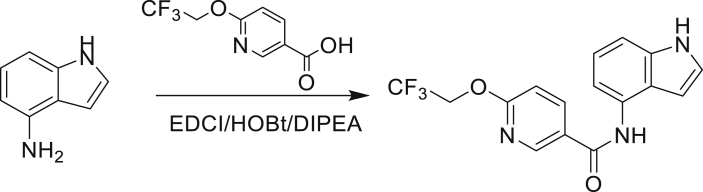

To a solution of 6-(2,2,2-trifluoroethoxy)pyridine-3-carboxylic acid (100 mg, 0.452 mmol and N-(1H-indol-4-yl)-6-(2,2,2-trifluoroethoxy)nicotinamide (71.7 mg, 0.542 mmol) in DMF (1 mL) was added EDCI (130 mg, 0.678 mmol), HOBt (91.6 mg, 0.678 mmol) and DIPEA (175 mg, 1.36 mmol) and the solution was stirred at 25°C for 16 hours. The mixture was poured into an ice-water (5 mL), the resulting precipitate collected by filtration, washed with water (2 mL×3) and dried under vacuum to give N-(1H-indol-4-yl)-6-(2,2,2-trifluoroethoxy)nicotinamide (120 mg, 79.2%) as a black solid. LRMS (ESI) m/z 336.0 [M + H]+

###### Step 2

**Figure:**
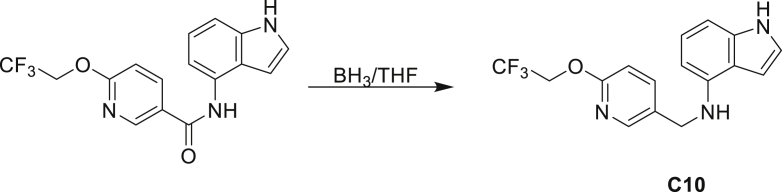

To a solution of N-(1H-indol-4-yl)-6-(2,2,2-trifluoroethoxy)nicotinamide (100 mg, 0.298 mol) in THF (1 mL) was added BH_3_•THF (1 M, 0.596 mL) at 25°C and the solution was stirred at 50°C for 3 hours. The reaction mixture was concentrated *in vacuo* to give a crude residue which was azetroped with EtOAc (2 mL×3). The crude product was purified by Prep-HPLC (Method F). to give the desired compound **C10** (27.3 mg, 27.6% yield) as a gray solid. ^1^H NMR (400 MHz, DMSO-d6) δ 10.82 (br s, 1H), 8.21 (d, J=2.0 Hz, 1H), 7.80 (dd, J=2.4, 8.5 Hz, 1H), 7.12 - 7.08 (m, 1H), 6.95 - 6.86 (m, 1H), 6.83 - 6.74 (m, 1H), 6.69 - 6.58 (m, 2H), 6.23 (t, J=6.2 Hz, 1H), 6.00 (d, J=7.6 Hz, 1H), 4.95 (q, J=9.2 Hz, 2H), 4.38 (d, J=6.2 Hz, 2H), 1.11 - 0.99 (m, 1H); LRMS (ESI) m/z 322.1 [M + H]+

##### N-(6-(2,2,2-trifluoroethoxy)pyridine-3-yl)-1H-indole-4-carboxamide (**C11**)

###### Step 1

**Figure:**
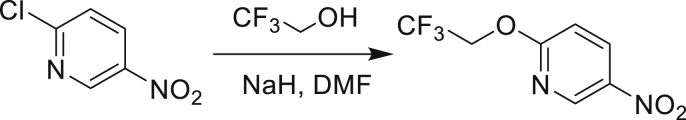

To a mixture of sodium hydride (45.4 mg, 1.14 mmol, 60% w/w) in DMF (10 mL) was added 2,2,2-trifluoroethanol (100 mg, 999 umol, 1.06 eq) at 0°C and the mixture was stirred at 0°C for 1 hour. A solution of 2-chloro-5-nitropyridine (150 mg, 0.946 mmol) in DMF (10 mL) was added to the cooled reaction mixture at 0°C and the mixture was stirred at 25°C for 2 hours. The reaction was concentrated in vacuum to remove most of the solvent. The residue was poured into saturated of ammonium chloride solution (200 mL) and stirred for 5 min. The aqueous phase was extracted with EtOAc (30 mL×3). The combined organic phases were washed with brine (20 mL), dried with anhydrous anhydrous sodium sulfate, filtered and concentrated *in vacuo* to give 5-nitro-2-(2,2,2-trifluoroethoxy)pyridine (120 mg, 54.1% yield) as a white solid. ^1^H NMR (400MHz, DMSO-d_6_): δ 9.12 (d, J=2.8 Hz, 1H), 8.59 (dd, J=2.8, 9.2 Hz, 1H), 7.25 (d, J=9.2 Hz, 1H), 5.16 (q, J=9.2 Hz, 2H); LRMS (ESI) m/z 223.0 [M + H]+

###### Step 2

**Figure:**
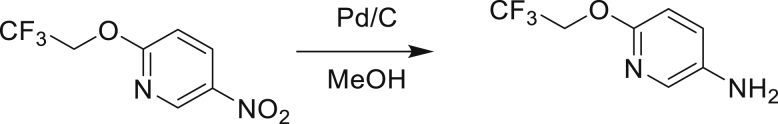

To a solution of 5-nitro-2-(2,2,2-trifluoroethoxy)pyridine (120 mg, 0.540 mmol) in MeOH (10 mL) was added 10% Pd/C (40 mg) under H_2_. The suspension was degassed under vacuum and purged with H_2_ several times. The mixture was stirred under H_2_ (15 psi) at 20°C for 4 hours. The reaction was filtered and the filtrate concentrated *in vacuo* to give 6-(2,2,2-trifluoroethoxy)pyridin-3-amine (100 mg, 96.3% yield) as a yellow oil. ^1^H NMR (400MHz, DMSO-d6): δ 7.51 (d, J=2.8 Hz, 1H), 7.07 (dd, J=2.8, 8.8 Hz, 1H), 6.69 (d, J=8.8 Hz, 1H), 4.93 (s, 2H), 4.80 (q, J=9.2 Hz, 2H)

###### Step 3

**Figure:**
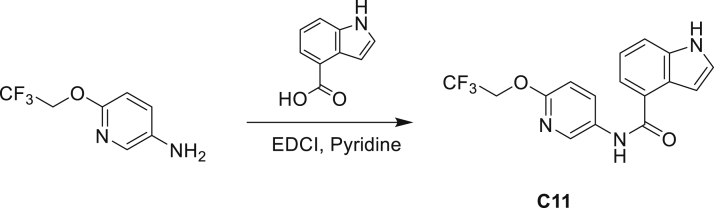

To a mixture of 6-(2,2,2-trifluoroethoxy)pyridin-3-amine (100 mg, 0.520 mmol) and 1H-indole-4-carboxylic acid (83.8 mg, 0.520 mmol) in pyridine (10 mL) was added EDCI (299 mg, 1.56 mmol) in one portion at 25°C, the reaction warmed to 45°C and stirred for 10 hours. The reaction was concentrated *in vacuo* to remove most of the solvent, the residue was poured into water (20 mL) and stirred for 5 minutes. The aqueous phase was extracted with EtOAc (30 mL×3), the combined organic phases were washed with brine (20 mL), dried with anhydrous sodium sulfate, filtered and concentrated *in vacuo*. The residue was purified by prep-HPLC (Method B) to give the desired compound **C11** (74.2 mg, 42.5% yield) as a white solid. ^1^H NMR (400MHz, DMSO-d6): δ 11.41 (br s, 1H), 10.31 (s, 1H), 8.62 (d, J=2.5 Hz, 1H), 8.21 (dd, J=2.6, 8.9 Hz, 1H), 7.64 (d, J=8.0 Hz, 1H), 7.59 (d, J=7.2 Hz, 1H), 7.50 (t, J=2.7 Hz, 1H), 7.23 (t, J=7.7 Hz, 1H), 7.03 (d, J=8.8 Hz, 1H), 6.91 - 6.81 (m, 1H), 4.99 (q, J=9.2 Hz, 2H); LRMS (ESI) m/z 336.0 [M + H]+

##### N-(indolin-4-yl)-6-(2,2,2-trifluoroethoxy)nicotinamide (**C12**)

###### Step 1

**Figure:**
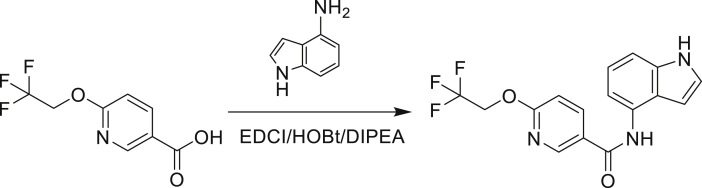

EW

To a solution of 6-(2,2,2-trifluoroethoxy)nicotinic acid (100 mg, 0.452 mmol) and 1H-indol-4-amine (71.7 mg, 0.542 mmol) in DMF (1 mL) was added EDCI (130 mg, 0.678 mmol), HOBt (91.6 mg, 0.678 mmol) and DIPEA (175 mg, 1.36 mmol and the solution was stirred at 25°C for 20 hours. The mixture was poured into an ice-water (5 mL), the resulting precipitate collected by filtration, washed with water (2 mL×3) and dried under vacuum to give N-(1H-indol-4-yl)-6-(2,2,2-trifluoroethoxy)nicotinamide (140 mg, 87.90% yield) was obtained as a black solid. LRMS (ESI) m/z 336.0 [M + H]+

###### Step 2

**Figure:**
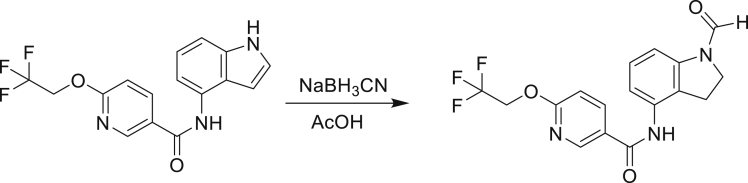

N-(1H-indol-4-yl)-6-(2,2,2-trifluoroethoxy)nicotinamide (140 mg, 0.417 mmol) in AcOH (1 mL) was added sodium cyanoborohydride (52.4 mg, 0.835 mmol) and the reaction was stirred at 25°C for 4 hours. The mixture was poured into water (1 mL) and extracted with EtOAc (15 mL×4). The combined organic layers were dried over anhydrous sodium sulfate and concentrated *in vacuo*. The crude product was purified by Prep-HPLC (Method D) to give N-(1-formylindolin-4-yl)-6-(2,2,2-trifluoroethoxy)nicotinamide (120 mg, 0.328 mmol)*.* (ESI) m/z 366.0 [M + H]+

###### Step 3

**Figure:**
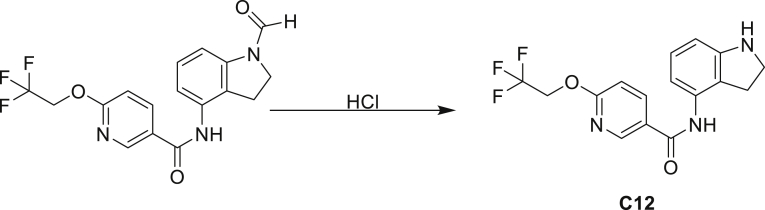

To a solution of N-(1-formylindolin-4-yl)-6-(2,2,2-trifluoroethoxy)nicotinamide (120 mg, 0.328 mmol in water (0.5 mL) and EtOH (0.5 mL) was added aq.HCl (4N, 1 mL) and the mixture was stirred for 6 hrs at 80°C. The mixture was cooled to room temperature, poured into an ice-water (5 mL), the resulting precipitate collected by filtration, washed with water (2 mL×3) and dried under vacuum. The crude product was purified by Prep-HPLC (Method E) to give the desired compound **C12** (21.7 mg, 19.5% yield) as an orange solid. ^1^H NMR (400 MHz, DMSO-d_6_): δ 9.93 (s, 1H), 8.78 (d, J=2.1 Hz, 1H), 8.30 (dd, J=2.4, 8.6 Hz, 1H), 7.13 (d, J=8.6 Hz, 1H), 6.91 (t, J=7.8 Hz, 1H), 6.64 (d, J=7.8 Hz, 1H), 6.38 (d, J=7.6 Hz, 1H), 5.56 (s, 1H), 5.10 (q, J=9.0 Hz, 2H), 3.46 - 3.39 (m, 2H), 2.88 (t, J=8.5 Hz, 2H); LRMS (ESI) m/z 338.0 [M + H]+.

##### N-(1H-indol-4-yl)-N-methyl-6-(2,2,2-trifluoroethoxy)nicotinamide (**C13**)

###### Step 1

**Figure:**
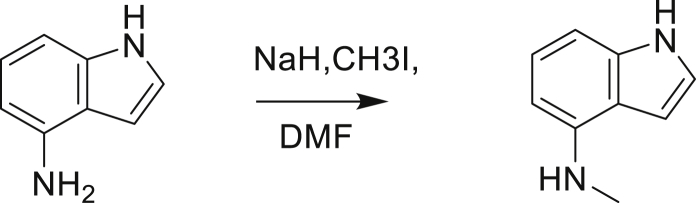

To a mixture of 1H-indol-4-amine (200 mg, 1.51 mmol) in DMF (10 mL) was added sodium hydride (72.63 mg, 1.82 mmol, 60% w/w) in one portion at 0°C and the mixture was stirred at 0°C for 1 hour. Methyl iodide (257.75 mg, 1.82 mmol) was added into the mixture which was stirred at 0°C for 1 hour. The reaction was concentrated *in vacuo* to remove most of the solvent to give a residue which was poured into water (20 mL) and stirred for 5 min. The aqueous phase was extracted with EtOAc (30 mL∗3). The combined organic phases were washed with brine (20 mL), dried with anhydrous anhydrous sodium sulfate, filtered and concentrated *in vacuo* to give N-methyl-1H-indol-4-amine (106 mg, 47.9% yield) as a white solid. LRMS (ESI) m/z 147.1 [M + H]+.

###### Step 2

**Figure:**
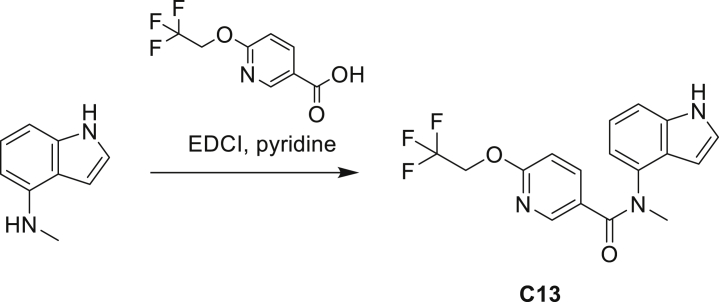

To a mixture of compound 6-(2,2,2-trifluoroethoxy)nicotinic acid (100 mg, 0.452 mmol) and N-methyl-1H-indol-4-amine (66.1 mg, 0.452 mmol) in pyridine (5 mL) was added EDCI (260 mg, 1.36 mmol) in one portion at 25°C, the mixture was warmed to 45°C and stirred for 10 hours. The reaction was concentrated *in vacuo* to remove most of the solvent to give a crude residue which was poured into water (20 mL) and stirred for 5 min. The aqueous phase was extracted with EtOAc (30 mL∗3). The combined organic phases were washed with brine (20 mL), dried with anhydrous sodium sulfate, filtered and concentrated *in vacuo*. The residue was purified by prep-HPLC (Method F) to give the desired compound **C13** (57.8 mg, 36.6% yield) as white solid. ^1^H NMR **(**400MHz, DMSO-*d*_*6*_**):** δ 10.22 (s, 1H), 8.85 (d, J=2.0 Hz, 1H), 8.37 (dd, J=2.4, 8.8 Hz, 1H), 7.43 (d, J=7.6 Hz, 1H), 7.33 - 7.28 (m, 2H), 7.19 - 7.11 (m, 2H), 6.61 (s, 1H), 5.12 (q, J=9.2 Hz, 2H), 3.81 (s, 3H); LRMS (ESI) m/z 350.1 [M + H]+.

##### N-(1H-benzo[d]imidazol-4-yl)-5-(2,2,2-trifluoroethoxy)picolinamide (**C14**)

**Figure:**
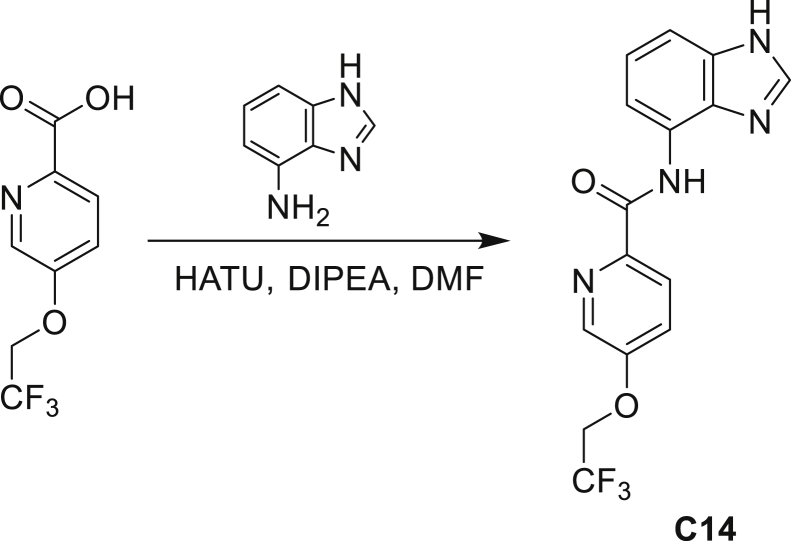

To a solution of 5-(2,2,2-trifluoroethoxy)picolinic acid (166 mg, 0.751 mmol) and 1H-benzimidazol-4-amine (100 mg, 0.751 mmol) in DMF (2 mL) was added HATU (571 mg, 1.50 mmol) and DIPEA (485.32 mg, 3.76 mmol) and the solution was stirred at 40°C for 16 hours. The mixture was poured into an ice-water (5 mL), the resulting precipitate collected by filtration, washed with water (2 mL×3) and dried under vacuum. The crude product was purified by Prep-HPLC (Method H) to give the desired product **C14** (18.2 mg, 7.1% yield) as a grey solid. ^1^H NMR (400 MHz, DMSO-d6) δ 10.85 (s, 1H), 8.61 (br d, J=2.4 Hz, 1H), 8.28 - 8.18 (m, 3H), 7.82 (dd, J=2.8, 8.7 Hz, 1H), 7.32 - 7.19 (m, 2H), 5.05 (q, J=8.6 Hz, 2H); LRMS (ESI) m/z 337.1 [M + H]+.

##### N-(1H-pyrrolo[2,3-b]pyridin-4-yl)-5-(2,2,2-trifluoroethoxy)picolinamide (**C15**)

###### Step 1

**Figure:**
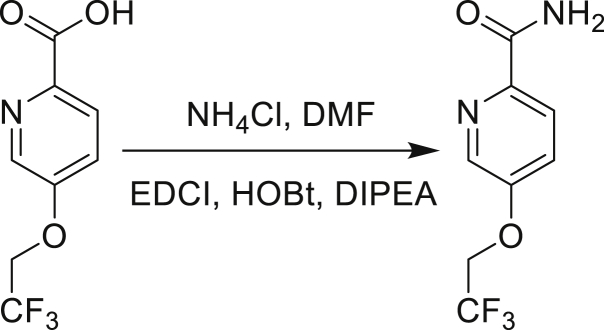

To the solution of 5-(2,2,2-trifluoroethoxy)picolinic acid (400 mg, 1.81 mmol) and ammonium chloride (194 mg, 3.62 mmol) in DMF (4 mL) was added EDCI (520 mg, 2.71 mmol), HOBt (366.63 /mg, 2.71 mmol) and DIPEA (701 mg, 5.43 mmol) and the reaction mixture was stirred at 25°C for 12 hours. The reaction was quenched with water (4 mL) and extracted with EtOAc (8 mL∗3). The combined organic layers were dried over anhydrous sodium sulfate and concentrated *in vacuo* to give 5-(2,2,2-trifluoroethoxy)picolinamide (300 mg, 75.3% yield) as pink solid. ^1^H NMR (400 MHz, DMSO-d6) δ 8.42 (d, J=2.8 Hz, 1H), 8.04 (d, J=8.8 Hz, 2H), 7.68 (dd, J=2.9, 8.7 Hz, 1H), 7.54 (br s, 1H), 4.98 (q, J=8.8 Hz, 2H); LRMS (ESI) m/z 221.0 [M + H]+.

###### Step 2

**Figure:**
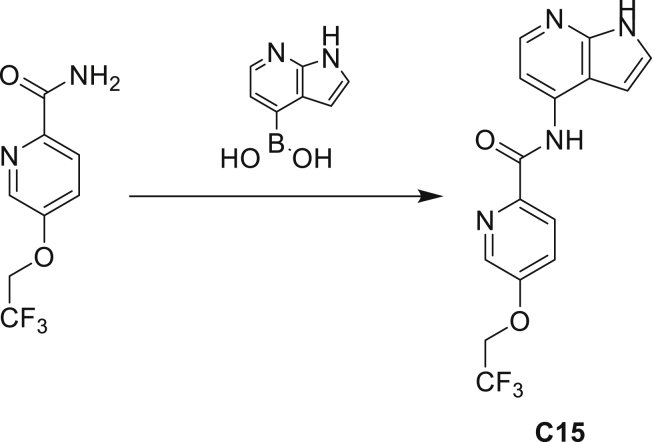

To the solution of 1H-pyrrolo[2,3-b]pyridin-4-ylboronic acid (147 mg, 0.908 mmol) and 5-(2,2,2-trifluoroethoxy)picolinamide (100 mg, 0.454 mmol) in DCM (2 mL) was added copper (II) acetate (165 mg, 0.908 mmol) and pyridine (108 mg, 1.36 mmol) and the reaction mixture was stirred at 25°C for 12 hours. The reaction mixture was concentrated *in vacuo* to give a crude residue which was purified by Prep-HPLC (Method I) to give the desired product **C15** (1.6 mg, 0.97% yield as yellow solid. ^1^H NMR (400 MHz, DMSO-d6) δ 11.73 (br s, 1H), 10.47 (s, 1H), 8.59 (d, J=2.9 Hz, 1H), 8.26 - 8.19 (m, 2H), 7.88 (d, J=5.3Hz, 1H), 7.82 (dd, J=2.8, 8.8 Hz, 1H), 7.49 - 7.44 (m, 1H), 6.63 (d, J=1.3 Hz, 1H), 5.09 - 5.03 (m, 2H)**;** LRMS (ESI) m/z 337.1 [M + H]+.

##### N-(1H-indol-4-yl)-6-(2,2,2-trifluoroethoxy)pyridazine-3-carboxamide (**C16**)

###### Step 1

**Figure:**
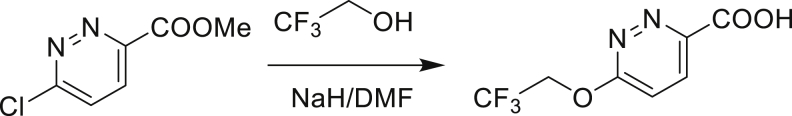

To a suspension of sodium hydride (174 mg, 4.35 mmol, 60% w/w) in DMF (1 mL) was added 2,2,2-trifluoroethanol (348 mg, 3.48 mmol) dropwise at 0°C and the mixture was stirred at 0°C for 30 minutes. Methyl 6-chloropyridazine-3-carboxylate (500 mg, 2.90 mmol) in DMF (1 mL) was added dropwise at 0°C and the mixture was stirred at 50°C for 2.5 hours. The mixture was poured into an ice-water (1 mL) and extracted with EtOAc (1 mL∗3). The combined organic layer was washed with brine (1 mL∗3), dried over anhydrous sodium sulfate, filtered and the filtrate was concentrated *in vacuo* to give 6-(2,2,2-trifluoroethoxy)pyridazine-3-carboxylic acid (390 mg, 60.6% yield) as a yellow solid. LRMS (ESI) m/z 223.0 [M + H]+.

###### Step 2

**Figure:**
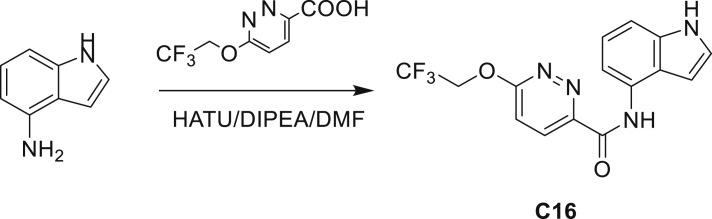

To a solution of 6-(2,2,2-trifluoroethoxy)pyridazine-3-carboxylic acid (390 mg, 1.76 mmol) and 1H-indol-4-amine (278 mg, 2.11 mmol) in DMF (2 mL) was added HATU (1.34 g, 3.51 mmol) and DIPEA (1.13 g, 8.78 mmol) and the solution was stirred at 25°C for 4 hours. The mixture was poured into an ice-water (5 mL), the resulting precipitate collected by filtration, washed with water (2 mL×3) and dried under vacuum. The crude product was purified by Prep-HPLC (Method J) to give the desired product **C16** (24.1 mg, 4.1% yield) as a grey solid. ^1^H NMR (400 MHz, DMSO-d6) δ 11.26 (br s, 1H), 10.49 (s, 1H), 8.34 (d, J=9.0 Hz, 1H), 7.70 - 7.62 (m, 2H), 7.38 (t, J=2.8 Hz, 1H), 7.28 (d, J=8.2 Hz, 1H), 7.16 - 7.09 (m, 1H), 6.54 (br s, 1H), 5.34 (q, J=8.9 Hz, 2H); **L**RMS (ESI) m/z 337.0 [M + H]+.

##### *N*-(1H-indol-4-yl)-2-(p-tolyloxy)acetamide (**C17**)

**Figure:**
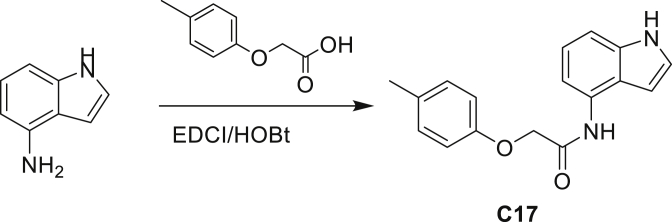

To a stirred solution of 2-(p-tolyloxy)acetic acid (0.183g, 1.1 mmol) and HOBt (0.203g, 1.5 mmol) in DMSO (2 mL) was added 4-aminoindole (0.132g, 1 mmol) and the reaction stirred for 30 minutes. EDCI (0.186g, 1.2 mmol) was added and stirring continued for 24 hours. The crude reaction mixture was purified by preparative HPLC (Method K), to give the desired product (**C17**) (0.103g, 36.8%) as an off-white solid. ^1^H NMR (400 MHz, DMSO) δ 11.13 (s, 1H), 9.65 (s, 1H), 7.53 (d, J=7.6 Hz, 1H), 7.31 (dd, J=2.8, 2.8 Hz, 1H), 7.16 (dd, J=8.2, 26.6 Hz, 3H), 7.03 (dd, J=7.9, 7.9 Hz, 1H), 6.94 (d, J=8.5 Hz, 2H), 6.59 (s, 1H), 4.76 (s, 2H), 2.25 (s, 3H); HRMS (ESI) m/z 281.1308 [M + H]+.

##### 2-(cyclopropylmethoxy)-N-(1H-indol-4-yl)acetamide (**C18**)

**Figure:**
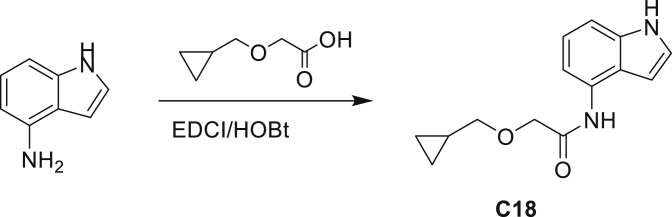

Using a similar method to **C17**, except using 2-(cyclopropylmethoxy)acetic acid (0.143g, 1.1 mmol), the desired compound (**C18**) (0.089g, 36.5%) was obtained as an off-white solid. ^1^H NMR (400 MHz, DMSO) d 10.90 (s, 1H), 9.00 (s, 1H), 7.34 (d, J=7.7 Hz, 1H), 7.08 (dd, J=2.8, 2.8 Hz, 1H), 6.94 (d, J=8.2 Hz, 1H), 6.79 (dd, J=7.9, 7.9 Hz, 1H), 6.28 (s, 1H), 3.90 (s, 2H), 3.19 (d, J=6.9 Hz, 2H), 0.93 - 0.84 (m, 1H), 0.31 - 0.25 (m, 2H), 0.03 - 0.00 (m, 2H); HRMS (ESI) m/z 245.1300 [M + H]+.

##### (1s,3s)-N-(1H-indol-4-yl)-3-phenoxycyclobutane-1-carboxamide (C19)

###### Step 1

**Figure:**
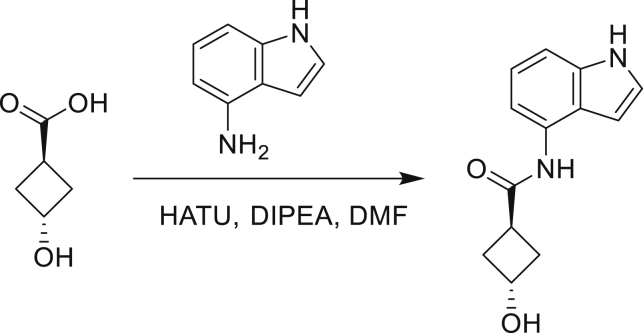

To a solution of (1r,3r)-3-hydroxycyclobutane-1-carboxylic acid (300 mg, 2.58 mmol) and 1H-indol-4-amine (410 mg, 3.10 mmol) in DMF (3 mL) was added HATU (1.96 g, 5.17 mmol) and DIPEA (1.67 g, 12.92 mmol) and the mixture was stirred at 40°C for 16 hours. The reaction was quenched with water (6 mL) and extracted with EtOAc (12 mL∗3). The combined organic layers were dried over anhydrous sodium sulfate and concentrated *in vacuo*. The crude residue was purified by Reverse phase flash column (water/MeCN+ 0.1% FA) to give (1r,3r)-3-hydroxy-N-(1H-indol-4-yl)cyclobutane-1-carboxamide (400 mg, 67.4% yield) as a pink solid. ^1^H NMR (400 MHz, DMSO-d6) δ 11.18 - 11.00 (m, 1H), 9.43 (s, 1H), 7.61 (d, J=7.6 Hz, 1H), 7.35 - 7.24 (m, 1H), 7.17 - 7.10 (m, 1H), 7.04 - 6.97 (m, 1H), 6.68 (br s, 1H), 5.16 (d, J=7.1 Hz, 1H), 4.11 - 3.92 (m, 1H), 2.89 - 2.75 (m, 1H), 2.44 - 2.30 (m, 2H), 2.44 - 2.30 (m, 1H), 2.14 - 1.98 (m, 2H); LRMS (ESI) m/z 231.1 [M + H]+.

###### Step 2

**Figure:**
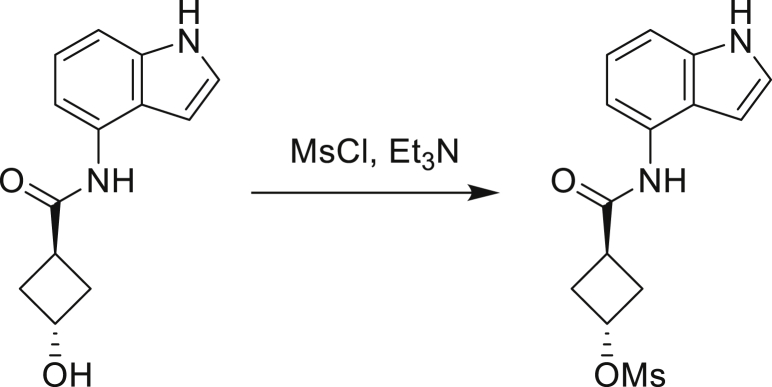

To a solution of (1r,3r)-3-hydroxy-N-(1H-indol-4-yl)cyclobutane-1-carboxamide (400 mg, 1.34 mmol) in DCM (4 mL) was added triethylamine (271 mg, 2.68 mmol) and methanesulfonyl chloride (307 mg, 2.68 mmol) and the mixture was stirred at 25°C for 12 hrs. The reaction was quenched with water (1 mL) and extracted with EtOAc (2 mL∗3). The combined organic layers were dried over anhydrous sodium sulfate and concentrated *in vacuo.* The crude residue was purified by Reverse phase flash column (water/MeCN+ 0.1% FA) to give (1r,3r)-3-((1H-indol-4-yl)carbamoyl)cyclobutyl methanesulfonate (250 mg, 60.5% yield) as gray solid. ^1^H NMR (400 MHz, DMSO-d6) δ 11.10 (br s, 1H), 9.63 (br s, 1H), 7.58 (br d, J=7.5 Hz, 1H), 7.28 (br s, 1H), 7.16 (br d, J= 8.4 Hz, 1H), 7.07 - 6.95 (m, 1H), 6.66 (br s, 1H), 4.99 (br s, 1H), 3.22 - 3.05 (m, 3H), 2.67 (br s, 1H), 2.34 (br s, 4H); LRMS (ESI) m/z 309.0 [M + H]+.

###### Step 3

**Figure:**
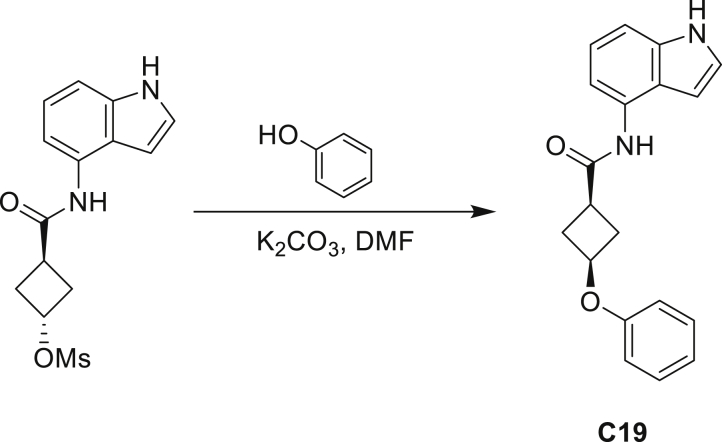

To a solution of phenol (114 mg, 1.22 mmol) in DMF (1.25 mL) was added cesium carbonate (528 mg, 1.62 mmol) and the mixture was stirred at 80°C for 1 hour. (1r,3r)-3-((1H-indol-4-yl)carbamoyl)cyclobutyl methanesulfonate (250 mg, 0.811 mmol) in DMF (1.25 mL) was then added and the reaction mixture stirred at 80°C for 1 hour. The reaction was quenched with water (2 mL) and extracted with EtOAc (3 mL∗3). The combined organic layers were dried over anhydrous sodium sulfate and concentrated *in vacuo*. The crude product was purified by Prep-HPLC (Method L) to give the desired product **C19** (4.2 mg, 1.7% yield) as a white solid. ^1^H NMR (400 MHz, DMSO-d6) δ 7.65 (d, J = 7.6 Hz, 1H), 7.33 - 7.26 (m, 3H), 7.14 (d, J = 8.1 Hz, 1H), 7.05 - 6.98 (m, 1H), 6.93 (t, J = 7.3 Hz, 1H), 6.84 (br d, J = 8.2 Hz, 2H), 6.70 (br s, 1H), 4.90 (br t, J = 6.2 Hz, 1H), 3.51 (td, J = 4.8, 9.4 Hz, 1H), 2.74 - 2.67 (m, 2H), 2.39 - 2.32 (m, 2H); LRMS (ESI) m/z 307.1 [M+H]+.

##### N-(1H-indol-4-yl)-6-(methyl(2,2,2-trifluoroethyl)amino)nicotinamide (**C20**)

###### Step 1

**Figure:**
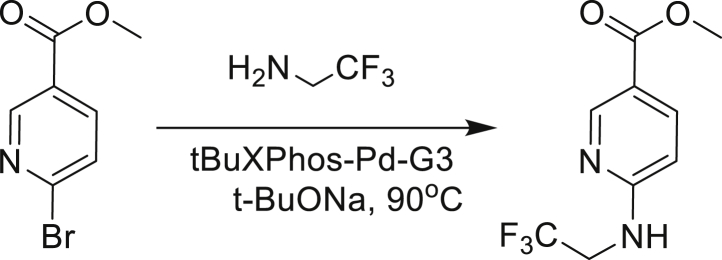

To the reaction mixture of methyl 6-bromonicotinate (600 mg, 3.50 mmol), 2,2,2-trifluoroethan-1-amine (520 mg, 5.25 mmol) and t-BuONa (2 M in THF, 5.25 mL, 3 eq) in dioxane (6 mL) was added tBuXPhos-Pd-G_3_ (277.78 mg, 349.69 umol, 0.1 eq) under N_2_ and the reaction was stirred at 90°C for 12 hrs. The reaction mixture was filtered and the filtrate was concentrated *in vacuo*. The residue was purified by column chromatography (SiO_2_, Petroleum Ether: EtOAc = 10:1 -5:1) to give methyl 6-((2,2,2-trifluoroethyl)amino)nicotinate (230 mg, 27.7% yield) as light yellow solid. ^1^H NMR (400MHz, CDCl3) δ 8.79 (d, J = 1.8 Hz, 1H), 8.05 (dd, J = 2.3, 8.7 Hz, 1H), 6.50 (d, J = 8.8 Hz, 1H), 5.03 (br s, 1H), 4.20 (dq, J = 6.8, 9.0 Hz, 2H), 3.89 (s, 3H); LRMS (ESI) m/z 235.1 [M+H]+.

###### Step 2

**Figure:**
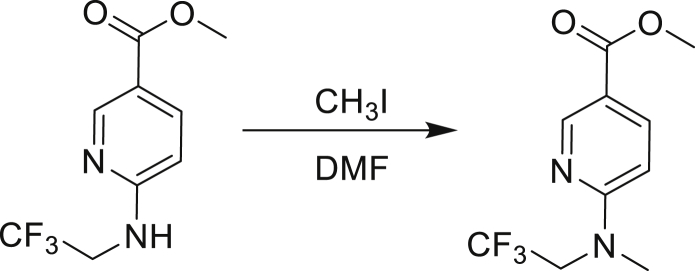

Methyl 6-((2,2,2-trifluoroethyl)amino)nicotinate (230 mg, 0.982 mmol), methyl iodide (558 mg, 3.93 mmol) and cesium carbonate (640 mg, 1.96 mmol) in DMF (2.5 mL) was stirred at 50°C for 3 hrs. The reaction mixture was poured into saturated ammonium chloride (5 mL) and extracted with EtOAc (2 mL∗3). The combined organic layer was washed with brine (5mL), dried over anhydrous sodium sulfate, filtered and the filtrate was concentrated *in vacuo* to give methyl 6-(methyl(2,2,2-trifluoroethyl)amino)nicotinate (220 mg, 86.5% yield) as a grey solid. ^1^H NMR (400MHz, CDCl3) δ 8.82 (d, J = 2.2 Hz, 1H), 8.11 (dd, J = 2.3, 8.9 Hz, 1H), 6.60 (d, J = 8.9 Hz, 1H), 4.42 (q, J = 9.0 Hz, 2H), 3.89 (s, 3H), 3.20 (s, 3H)); LRMS (ESI) m/z 249.1 [M+H]+.

###### Step 3

**Figure:**
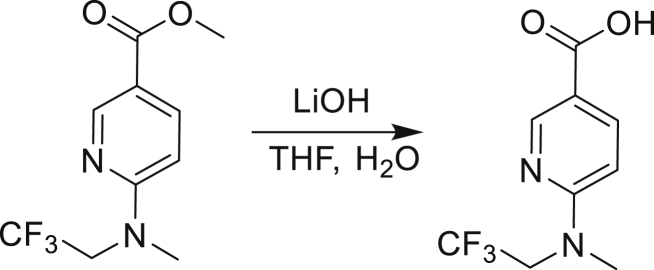

6-(methyl(2,2,2-trifluoroethyl)amino)nicotinate (120 mg, 0.483 mmol) and lithium hydroxide monohydrate (40.6 mg, 0.967 mmol) in THF (0.6 mL) and water (0.6 mL) was stirred at 25°C for 12 hrs. The reaction mixture was adjusted to pH=2 with aq.HCl (4M) and extracted with EtOAc (2 mL∗3). The combined organic layer was washed with brine (5 mL), dried over anhydrous sodium sulfate, filtered and the filtrate was concentrated to give 6-(methyl(2,2,2-trifluoroethyl)amino)nicotinic acid (100mg, 88% yield) as a grey solid. LRMS (ESI) m/z 235.1 [M+H]+.

###### Step 4

**Figure:**
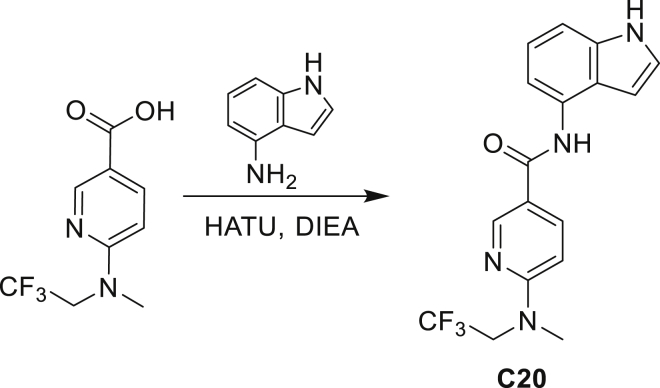

6-(methyl(2,2,2-trifluoroethyl)amino)nicotinic acid (100 mg, 427.03 umol, 1 eq), 1H-indol-4-amine (56.4 mg, 0.427 mmol), HATU (244 mg, 641 umol) and DIPEA (166 mg, 1.28 mmol) in DMF (1.5 mL) was stirred at 25°C for 12 hrs. The reaction mixture was concentrated *in vacuo* to give a crude residue which was purified by Prep-HPLC (Method L) to obtain the desired compound **C20** (48.8 mg, 32.8% yield) as a grey solid. ^1^H NMR (400MHz, DMSO) δ 11.10 (br s, 1H), 9.90 (s, 1H), 8.79 (d, J = 2.2 Hz, 1H), 8.19 (dd, J = 2.4, 8.9 Hz, 1H), 7.36 (d, J = 7.6 Hz, 1H), 7.29 (t, J = 2.8 Hz, 1H), 7.22 (d, J = 8.1 Hz, 1H), 7.09 - 7.03 (m, 1H), 6.91 (d, J = 8.9 Hz, 1H), 6.59 (d, J = 2.0 Hz, 1H), 4.59 (q, J = 9.6 Hz, 2H), 3.17 (s, 3H). LRMS (ESI) m/z 349.1 [M+H]+

##### N-(1H-indol-4-yl)-5-methylfuran-2-carboxamide (**21**)

**Figure:**
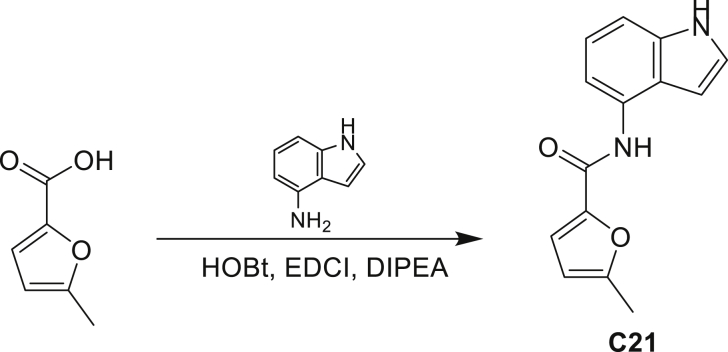

1H-indol-4-amine (100 mg, 0.757 mmol), 5-methylfuran-2-carboxylic acid (86.8 mg, 0.688 mmol), HOBt (139 mg, 1.03 mmol), EDCI (198 mg, 1.03 mmol) and DIPEA (267 mg, 2.06 mmol) in DMF (1 mL) was stirred at 25°C for 12 hours. The reaction mixture was concentrated *in vacuo* to give a crude residue which was purified by Prep-HPLC (Method L) to give the desired compound **C21** (41.4 mg, 0.172 mmol, 25.0% yield) as a grey solid. ^1^H NMR (400MHz, DMSO) δ 11.14 (br s, 1H), 9.74 (s, 1H), 7.33 - 7.23 (m, 4H), 7.11 - 7.03 (m, 1H), 6.53 (br s, 1H), 6.32 (d, J=2.7 Hz, 1H), 2.39 (s, 3H); LRMS (ESI) m/z 241.1 [M+H]+.

### Quantification and statistical analysis

Statistical analysis was performed in GraphPad Prism version 8.0.1 for Widnows, GraphPad Software, San Diego, Califonia USA, www.graphpad.com. The number of biological replicates and statistical tests applied for each experiment is given in the corresponding figure legend.

## Supplemental information

Document S1. Figures S1–S7 and Tables S1–S5

Document S2. Article plus supplemental information
