# Supplementary material for: Resistance of Mycobacterium tuberculosis to indole 4-carboxamides occurs through alterations in drug metabolism and tryptophan biosynthesis
Source: Cell Chem Biol. 2021 Aug 19;28(8):1180–1191.e20. doi: 10.1016/j.chembiol.2021.02.023 (PMC8379015; doi:10.1016/j.chembiol.2021.02.023)
Supplement: Document S1. Figures S1–S7 and Tables S1–S5 [file mmc1.pdf]

Cell Chemical Biology, Volume *28*

Supplemental information

Resistance of *Mycobacterium tuberculosis* to indole 4-carboxamides occurs through alterations in drug metabolism and tryptophan biosynthesis

M. Daben J. Libardo, Caroline J. Duncombe, Simon R. Green, Paul G. Wyatt, Stephen Thompson, Peter C. Ray, Thomas R. Ioerger, Sangmi Oh, Michael B. Goodwin, Helena I.M. Boshoff, and Clifton E. Barry III

Supplemental Text and Figures

Resistance of *Mycobacterium tuberculosis* to indole 4-carboxamides occurs through alterations in drug metabolism and alterations in tryptophan biosynthesis

M. Daben J. Libardo[1,5,7], Caroline J. Duncombe[1,7], Simon R. Green[2], Paul G. Wyatt[2], Stephen Thompson[2], Peter C. Ray[2,6], Thomas R. Ioerger[3], Sangmi Oh[1], Michael B. Goodwin[1], Helena I.M. Boshoff[1], Clifton E. Barry III[1,4,8]

[1]Tuberculosis Research Section, Laboratory of Clinical Immunology & Microbiology, National Institute of Allergy and Infectious Diseases, National Institutes of Health, Bethesda, MD 20892
[2]Drug Discovery Unit, Division of Biological Chemistry and Drug Discovery, School of Life Sciences, University of Dundee, UK, DD1 5EH
[3]Department of Computer Science and Engineering, Texas A&M University, College Station, TX 77843
[4] Institute for Infectious Disease and Molecular Medicine, University of Cape Town, Cape Town 7935, South Africa
[5]Present address: Infectious Disease & Vaccines, Merck Research Laboratories, Merck & Co. West Point, PA 19486
[6]Present address: Excentia Ltd., Oxfors OX1 3LD, UK
[7]these authors contributed equally
[8]Lead Contact

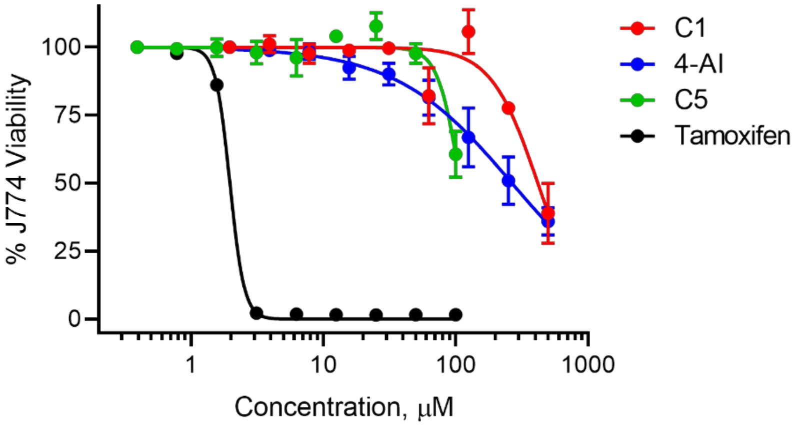

Figure S1. Cytotoxicity of indole-4-carboxamides and 4-aminoindole, related to Figure 1. Cytotoxicity of representative compounds in the study was measured using the CellTitre-Glo® luminescent cell viability assay. 10,000 J774 cells were seeded into wells of a 96-well plate and treated with the indicated compounds for 24 hrs. Data represent Mean ± SEM (n = 4).

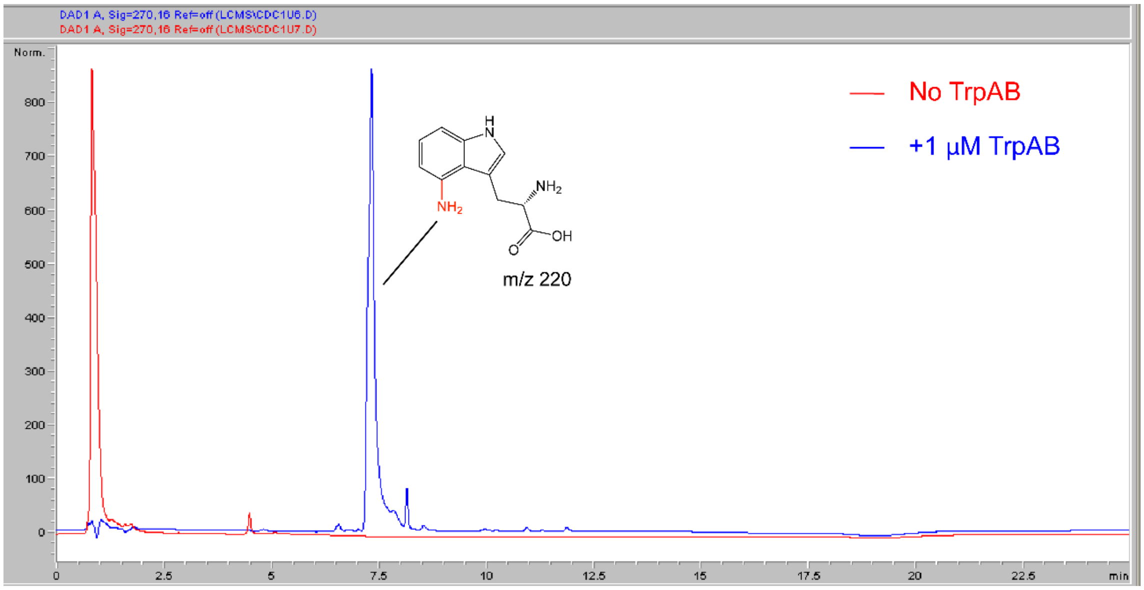

Figure S2. 4-Aminoindole is a substrate for TrpAB, related to Figure 6. 4-AI was incubated with 1μM TrpAB overnight before quenching the reaction with an equal volume of MeOH. A 2 μL aliquot of the reaction mixture was injected into the LC-MS to detect formation of 4-amino-L-Trp. Chromatograms are representative of three individual trials.

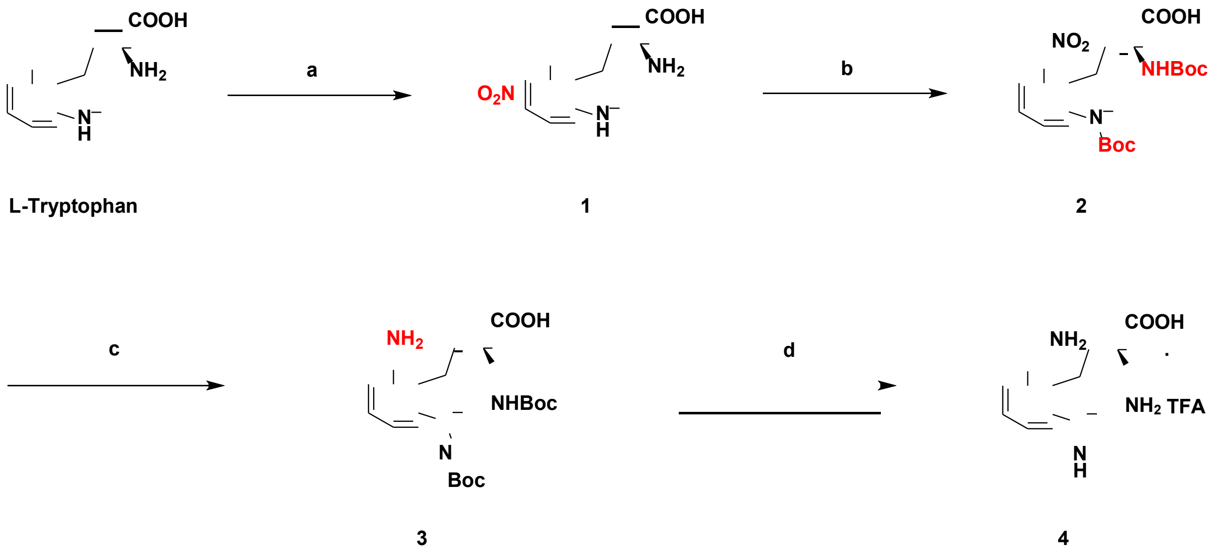

Figure S3. Chemical synthesis of 4-amino-L-Tryptophan, related to Figure 6. Reagents and conditions: (a) 70% HNO_3 (3 eq), AcOH, H_2O, rt, 4 h; (b) Boc anhydride (3 eq), DMAP (3 eq), NEt_3 (4 eq), DCM, rt, overnight; (c) Pd/C, H_2, rt, 3 h; (d) TFA, MC, rt, 2 h.

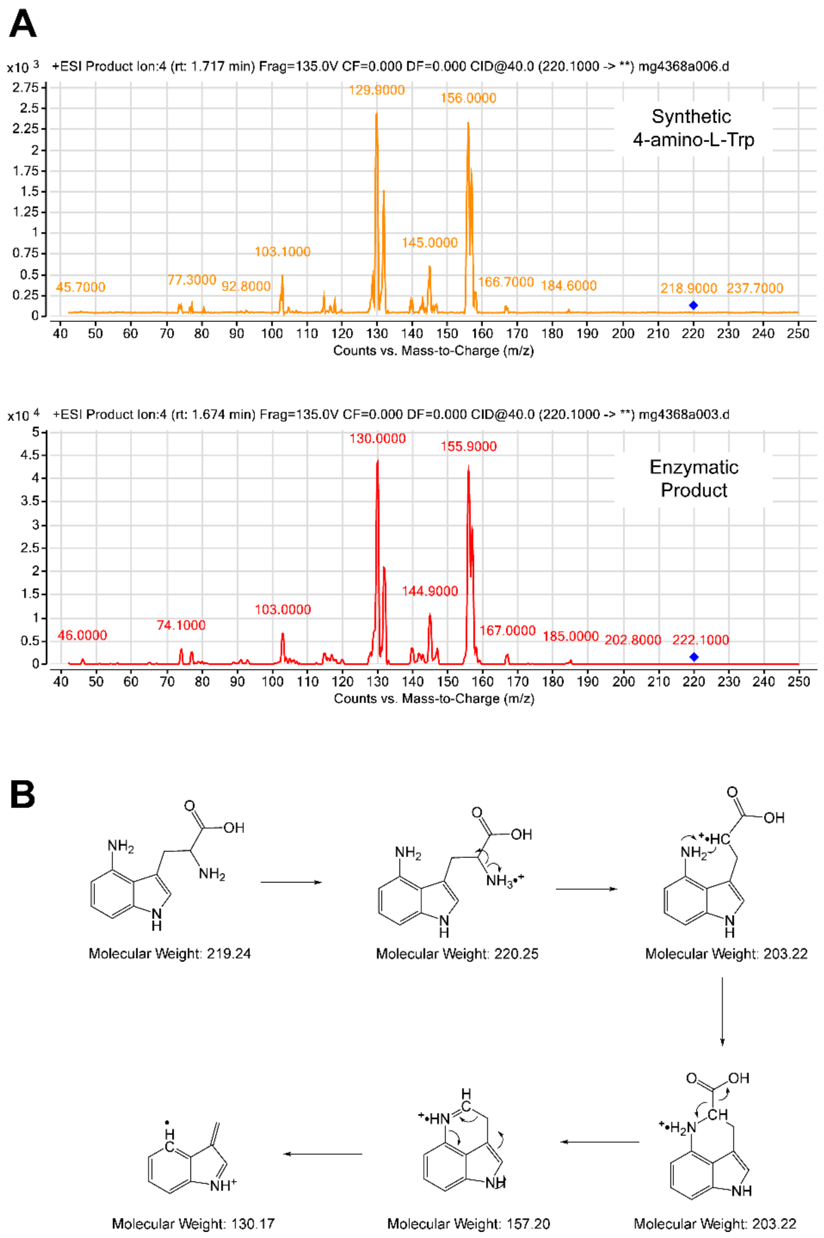

Figure S4. Fragmentation patterns of synthetic 4-amino-L-trp match the enzyme reaction product observed from 4-AI, related to Figure 6 (A) Triple quadrupole tandem MS fragmentation of synthetic 4-amino-L-Trp (top) and the enzymatic product obtained by incubating TrpAB with 4-AI (bottom). (B) Possible mechanism of fragmentation of 4-amino-L-Trp to account for the observed m/z in panel A and in Figure 6B and 6C.

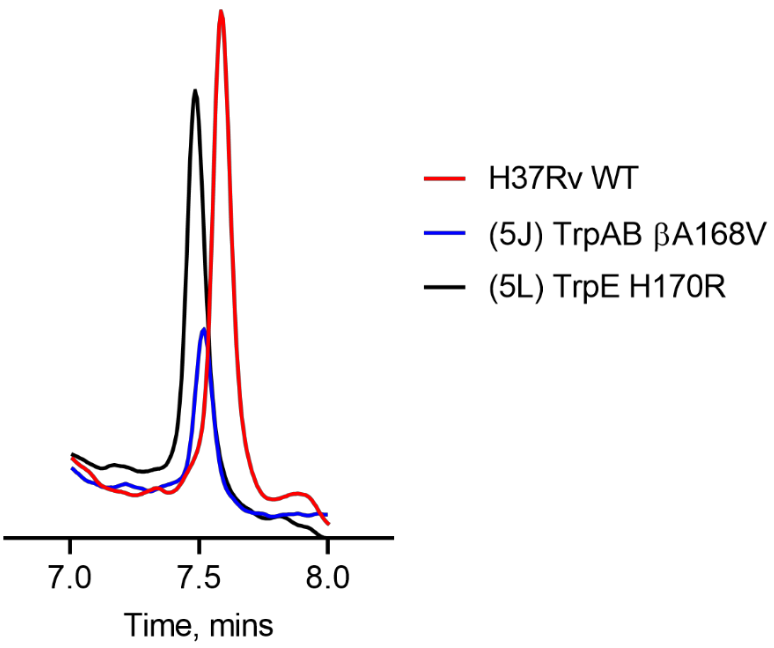

Figure S5. TrpAB mutant is less active than wild type in converting 4-AI to 4-a-Trp, Related to Figure 6. LC-MS chromatograms (270 nm) obtained when 4-AI was incubated with H37Rv WT or representative resistant mutants. The peak represent an m/z of 220.1 accounting for intracellular formation of 4-amino-L-Trp.

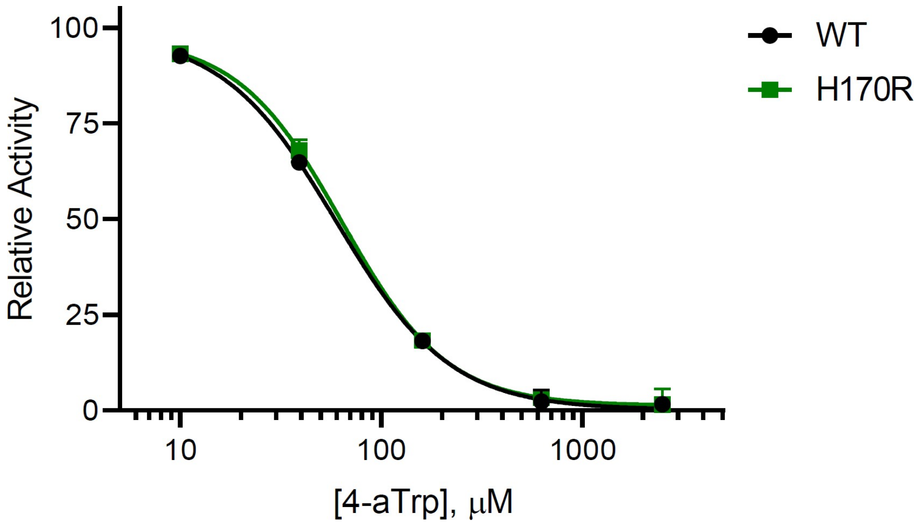

Figure S6. 4-Amino-Trp does not cause feedback inhibition of TrpE, Related to Figure 6. Inhibition of TrpE by 4-AI occurs with a high IC50 value of 60 μM. In addition, the mutant H170R which alleviates allosteric regulation by tryptophan, supporting that 4-aTrp does not inhibit TrpE.

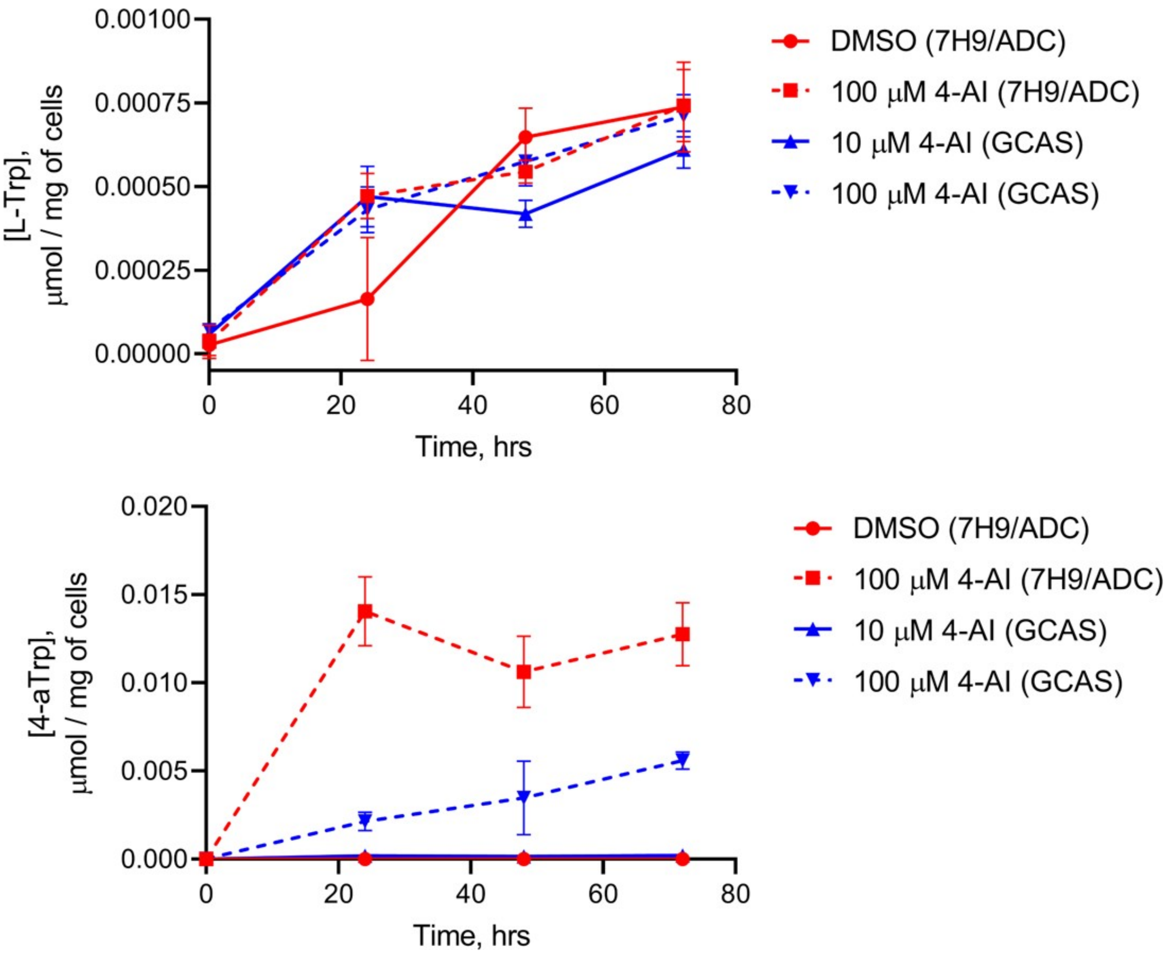

Figure S7. 4-AI incorporation into 4-a-Trp does not depress cellular levels of L-Trp, Related to Figure 6. Incorporation of 4-AI into whole cells of Mtb and the impact on intracellular L-tryptophan (top) and 4-aTrp (bottom) levels.

Table S1. Minimum Inhibitory Concentration (MIC) of Indole-4-carboxamides in various growth media against Mtb and other mycobacteria, related to Table 1.

Compound	MIC vs ***M. tuberculosis*** **H37Rv**, μM			MIC vs ***M. bovis BCG***, μM		MIC vs ***M. smegmatis*** **mc^{2}155**, μM
	7H9/ADC	7H9/GCas	GAST-Fe	7H9/ADC	7H9/GCas	7H9/GCas
**C1**	12.5	1.56	6.25	100	6.25	>100
**C2**	25	3.12	25	>100	12.5	>100
**C3**	>100	6.25	>100	>100	50	>100
**C4**	25	3.12	50	>100	12.5	>100

Table S2. Minimum Inhibitory Concentration (MIC) of 4-aminoindole and the corresponding free carboxylic acid building blocks of the NMMV03 compounds against Mtb H37Rv, related to Figure 2.

Compound	Structure	MIC, µM (7H9/GCas)
4-aminoindole	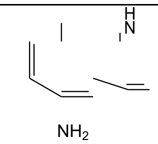	4.68
C1-COOH	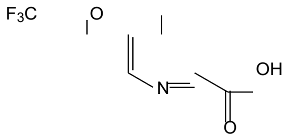	50
C2-COOH	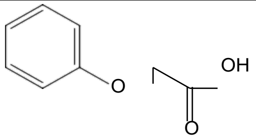	>100
C3-COOH	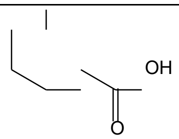	>100
C4-COOH	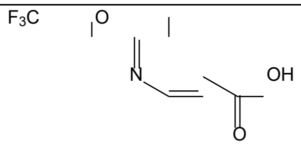	>100

Table S3. MIC of similar compounds lacking the 4-aminoindoleamide, related to Figure 2. Minimum Inhibitory Concentration (MIC) of representative members of the compound cluster against Mtb H37Rv.

Compound	Structure	MIC, µM (7H9/GCas)
C6	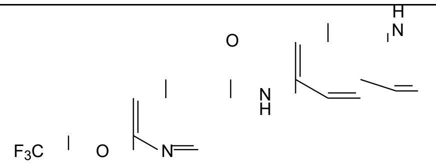	50
C7	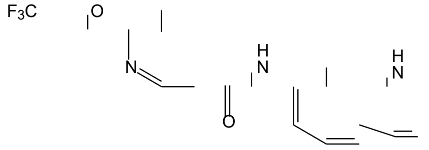	50
C8	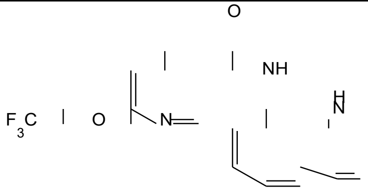	9.4
C9	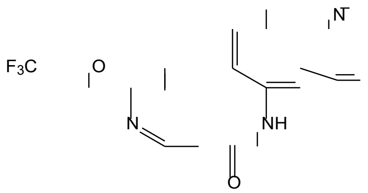	50
C10	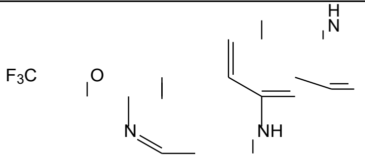	50
C11	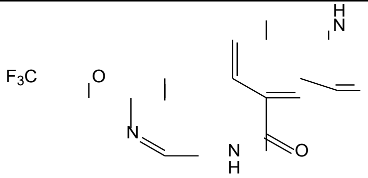	50
C12	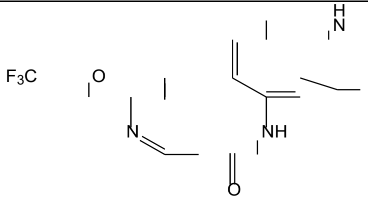	50

C13	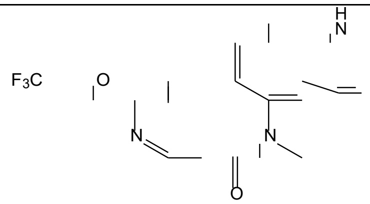	25
C14	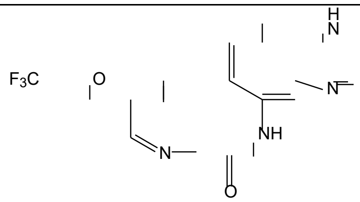	50
C15	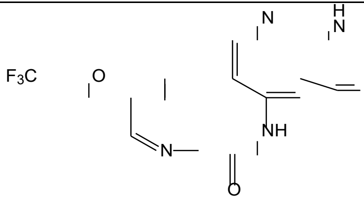	50
C16	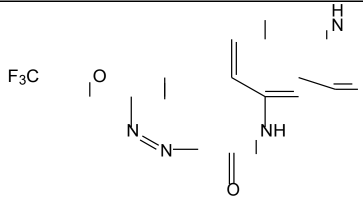	2.3
C17	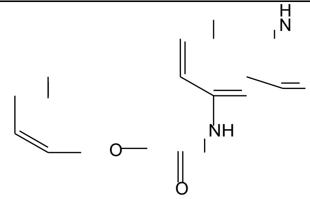	1.56
C18	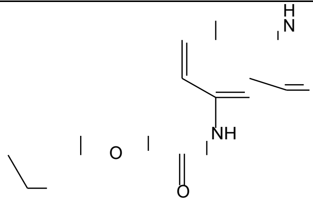	3.13
C19	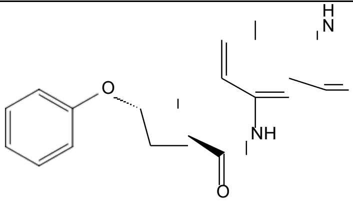	3.13
C20	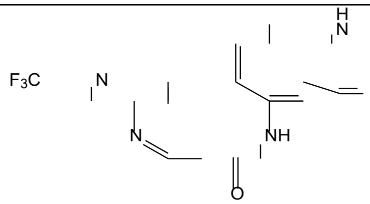	4.7

C21	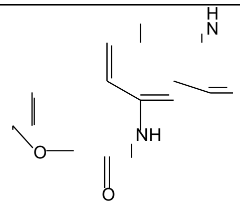	6.25

Table S4. C5-resistant mutants remain sensitive to indole-4-carboxamides, related to Figure 5. Mtb H37Rv C5^{R} mutant MIC against indole-4-carboxamides and the corresponding mutations found via whole genome sequencing.

**C5^{R} Strain**	**MIC, µM** (7H9/GCas)						
	**C1**	**C2**	**C3**	**C4**	**4-AI**	**C5**	**INH**
WT	3.12	6.25	12.5	6.25	6.25	0.39	0.16
A1 (*trpB* F188S)	1.56	3.12	12.5	3.12	6.25	>100	0.16
A2 (*trpB* Y200C)	6.25	6.25	12.5	6.25	6.25	>100	0.16
A4 (*trpA* P65L)	3.12	6.25	12.5	6.25	6.25	>100	0.16
B2 (*trpA* D136N)	3.12	6.25	12.5	6.25	6.25	>100	0.16

Table S5. Oligonucleotides used in this study, related to STAR methods.

Primers for Psmyc promoter swap	GTGTTCTAGAGGATCGTCGGCACC	P_{smyc}-F
	GCGCCCATGGAGATACCTCCTTAATTAAGCATGCGGATC	P_{smyc}-R
Primers for AmiC cloning	GCTACCATGGGCATGTCGCGCGTACACG	AmiC_pMV306-F
	GCTAATCGATCTACTCGGCGATATTTGGGGCGTGG	AmiC_pMV306-R
Primers for TrpB recombineering	GAGACCGGTGCCGGCCAGCACGGGGTCGCCACGGTCACCGCATGCGCATTGCTCGGCCTGGACTGTGTC	TrpB-A134V_Rec
	GCGCTAAACGTGGCCCGGATGCGATTGCTGGGTGTCGAAGTCGTCGCGGTTCAGACGGGCTCGAAAACG	TrpB-A168V_Rec
Primer for TrpE recombineering	CTGTTGCTGGCCACCGATGTGGCGGCGGTCGATCGCCACGAGGGCACCATCACGTTGATCGCCAACGCC	TrpE-H170R_Rec
Primer for RpsL recombineering	GCGGGCAACCTTCCGAAGCGCCGAGTTCGGCTTCCTCGGAGTGGTGGTGTACACGCGGGTGCATACACC	RpsL-K43R_Rec
Primers for confirming TrpB	CGATCTGGTTACCGCGGG	TrpB_RecCheck-F
	CCCGGTCGGCAAGTAACC	TrpB_RecCheck-R
Primers for confirming TrpE	GCCCGACTCCTTGCGC	TrpE_RecCheck-F
	CCGATGACCACCCAGGG	TrpE_RecCheck-R
Primers for confirming RpsL	AGTTTGAGGCAAGCTATG	RpsL_RecCheck-F
	CCCTTCAACAGAACCTTG	RpsL_RecCheck-R
Primers for TrpE overexpression	GCTACATATGCACGCCGACCTC	TrpE_pET28a-Exp-F
	GCTAAAGCTTTTAGCAGCCACTGCG	TrpE_pET28a-Exp-R
Primers for TrpE H170R mutagenesis	GCGGTCGATC**g**CCACGAGGGC	TrpE-H170R-F
	CGCCACATCGGTGGCCAG	TrpE-H170R-R
Primers for TrpB overexpression	GCTAAAGCTTATGAGTGCTGCCATC	TrpB_pRSFDuet-F
	GCTAAAGCTTTCAGTCGTTGCCCAG	TrpB_pRSFDuet-R
Primers for TrpA overexpression	GCTACATATGGTGGCGGTGGAAC	TrpA_pRSFDuet-F
	GCTACTCGAGTCATGCGGACATCC	TrpA_pRSFDuet-R
Primers for AmpR for TrpA cloning	CATGCCATGGGAATGGTGGCGGTGGAACAG	TrpA_AmpR-F
	CCCAAGCTTTCATGCGGACATCCCTAG	TrpA_AmpR-R
Primers for TrpB mutagenesis A168V	GTCGCCACGG**t**CACCGCATGC	TrpB-A168V_SDM-F
	CCCGTGCTGGCCGGCACC	TrpB-A168V_SDM-R
Primers for TrpB mutagenesis D261G	TTTCTCGATG**g**CCCAGGCGTA	TrpB-D261G_SDM-F
	CGCATGAAAAATACCAATGGC	TrpB- D261G_SDM-R
Primers for TrpCF expression	GCTACATATGATGATGCAAACCGTTTTAGC	Ec_TrpCF_Exp-F
	GCTAAAGCTTTTAATATGCGCGCAGCG	Ec_TrpCF_Exp-R
